# Tumor plasticity in macrophage niches promotes dormancy, EMT and chemoresistance of non-small cell lung cancer stem cells

**DOI:** 10.1038/s41420-026-03261-1

**Published:** 2026-07-31

**Authors:** Ann Zeuner, Giovanni Sette, Stefania Rossi, Elena Landolfo, Alessandro Vici, Rachele Rossi, Matilde Todaro, Alice Turdo, Vincenzo L. Lentini, Michele Signore, Alessandra Boe, Eleonora Petrucci, Maria Laura De Angelis, Federica Francescangeli

**Affiliations:** 1 Department of Oncology and Molecular Medicine, Istituto Superiore di Sanità, Rome, Italy; 2 Department of Health Promotion, Mother and Child Care, Internal Medicine and Medical Specialties (PROMISE), University of Palermo, Palermo, Italy; 3 Villa Sofia-Cervello Hospital, Palermo, Italy; 4 Core Facilities Technical-Scientific Service, Istituto Superiore di Sanità, Rome, Italy

**Keywords:** Non-small-cell lung cancer, Cancer microenvironment

## Abstract

Macrophages are the major immune cell type in the microenvironment of non-small cell lung cancer (NSCLC), playing a key role in tumor development, progression, and response to therapies. Elucidating the interplay between macrophages and NSCLC is crucial to fulfill the potential of macrophage-targeting strategies. We used NSCLC surgical specimens to generate multicellular spheroids enriched in cancer stem cells (CSCs) and highly responsive to microenvironmental cues. NSCLC spheroids were used to characterize state transitions induced by macrophage-tumor cell interactions in terms of proliferation, epithelial-to-mesenchymal transition (EMT), expression of stemness-related factors, self-renewal, and drug resistance. To define whether state transitions were induced by cell–cell contact or soluble factors, we compared direct co-cultures of spheroids and macrophages with transwell cultures, where cells are separated by a porous membrane. Cellular interactions were recorded by time-lapse microscopy and immunofluorescence (IF), whereas EMT, dormancy, stemness and chemoresistance were evaluated by real-time PCR, immunoblotting and functional assays. We found that co-cultures allowing cell–cell contact, but not transwell cultures, induced robust state transitions in NSCLC CSCs, promoting a state of dormancy associated with increased stemness, EMT and chemoresistance. In direct co-cultures (named NIDO, for Niche-Induced DOrmancy), macrophages and tumor cells formed nest-shaped aggregates and underwent a specular modulation of TGF-β levels, suggesting a physical transfer of this cytokine from macrophages to NSCLC cells within cellular nests. Treatment with Bafilomycin A1, an inhibitor of autophagosome-lysosome fusion and autolysosome acidification, inhibited TGF-β relocation and prevented the full spectrum of TGF-β-induced state transitions, including chemoresistance, EMT, and nest formation. By contrast, antibody-mediated TGF-β neutralization had only a partial effect, indicating secretory autophagy as a key mechanism underlying macrophage-tumor cell interplay. Altogether, these results show that lung macrophages crucially influence CSC phenotype, proliferation, and therapy resistance, pointing to a key role of local cell circuits in regulating tumor cell plasticity.

**Figure:**
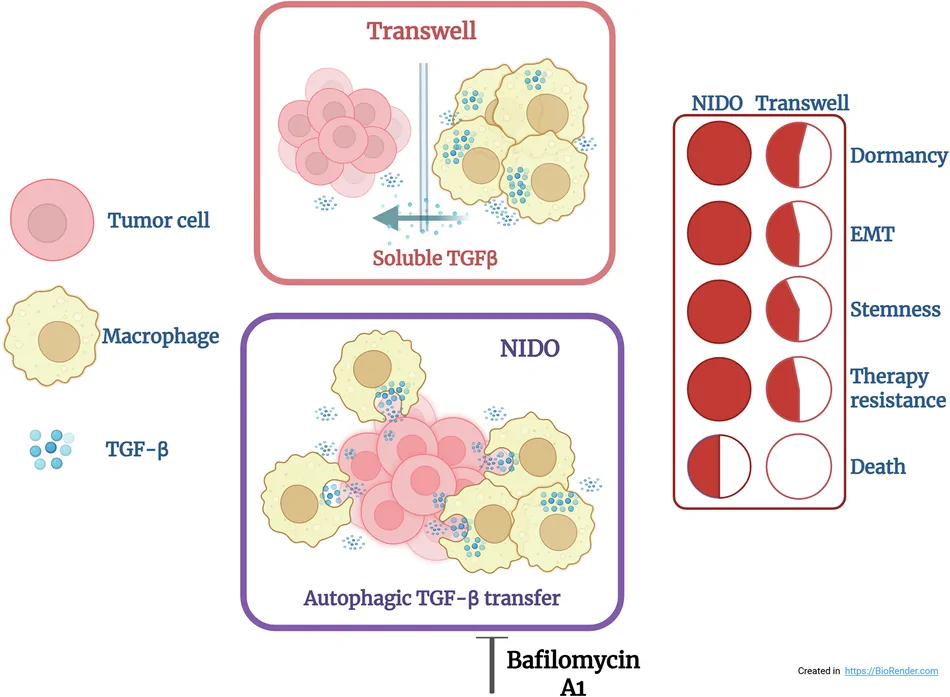

## Introduction

Lung cancer accounts for ~2.5 million new cases and ~1.8 million deaths yearly, representing the leading cause of cancer mortality [1]. Non-small cell lung cancer (NSCLC) represents ~85% of lung cancers, with its main histological subtypes being adenocarcinoma (AC) and squamous cell carcinoma (SCC) [2]. The interplay between NSCLC and the immune microenvironment is gaining increasing attention due to its implications for immunotherapy [3]. In healthy lungs, macrophages act as first-line sentinels against pathogens, also fulfilling a variety of functions related to tissue homeostasis and repair [4]. Macrophages also represent a major component of the NSCLC tumor microenvironment (TME), playing a role in tumor development, progression, immune escape, metastasis, and therapy resistance [5–8]. Tumor-associated macrophages (TAMs) were traditionally classified into M1 (pro-inflammatory/antitumorigenic) and M2 (anti-inflammatory/protumorigenic). However, TAMs are increasingly recognized as a spectrum of activation states displaying mixed M1/M2 features rather than being polarized in two extremes [9]. Currently, single-cell RNA sequencing coupled with multiplex immunofluorescence and spatial transcriptomics are providing new insights into TAMs’ nature [10–13]. The incorporation of immune cells into three-dimensional (3D) experimental models such as spheroids and organoids provides new opportunities to investigate therapeutically relevant cellular interactions. For example, co-cultures of NSCLC organoids and T cells have been recently employed to study tumor-immune system crosstalk [14, 15]. Research using NSCLC co-cultures with macrophages mostly relies on NSCLC commercial cell lines. However, these fail to reproduce tumor heterogeneity and are not reliable for preclinical drug testing [9]. Differently, 3D cultures generated from NSCLC surgical samples match tumors’ mutational profiles, mimic tumor heterogeneity, and can be used for personalized drug testing [9, 16]. We used cancer stem cells (CSCs)-enriched spheroids generated from NSCLC tumor specimens co-cultured with THP-1-derived macrophages to investigate CSCs-macrophage interactions. We found that physical contact, but not cytokine-mediated signaling, was essential to produce a full spectrum of state transitions in CSCs such as EMT, chemoresistance, dormancy, and stemness-related properties. These observations provide new insights into the role in NSCLC, suggesting that cell–cell interactions should be taken into consideration in future therapeutic strategies aimed at macrophage targeting.

## Results

### Phenotypic and molecular characterization of NSCLC spheroids

**Fig. 1:**
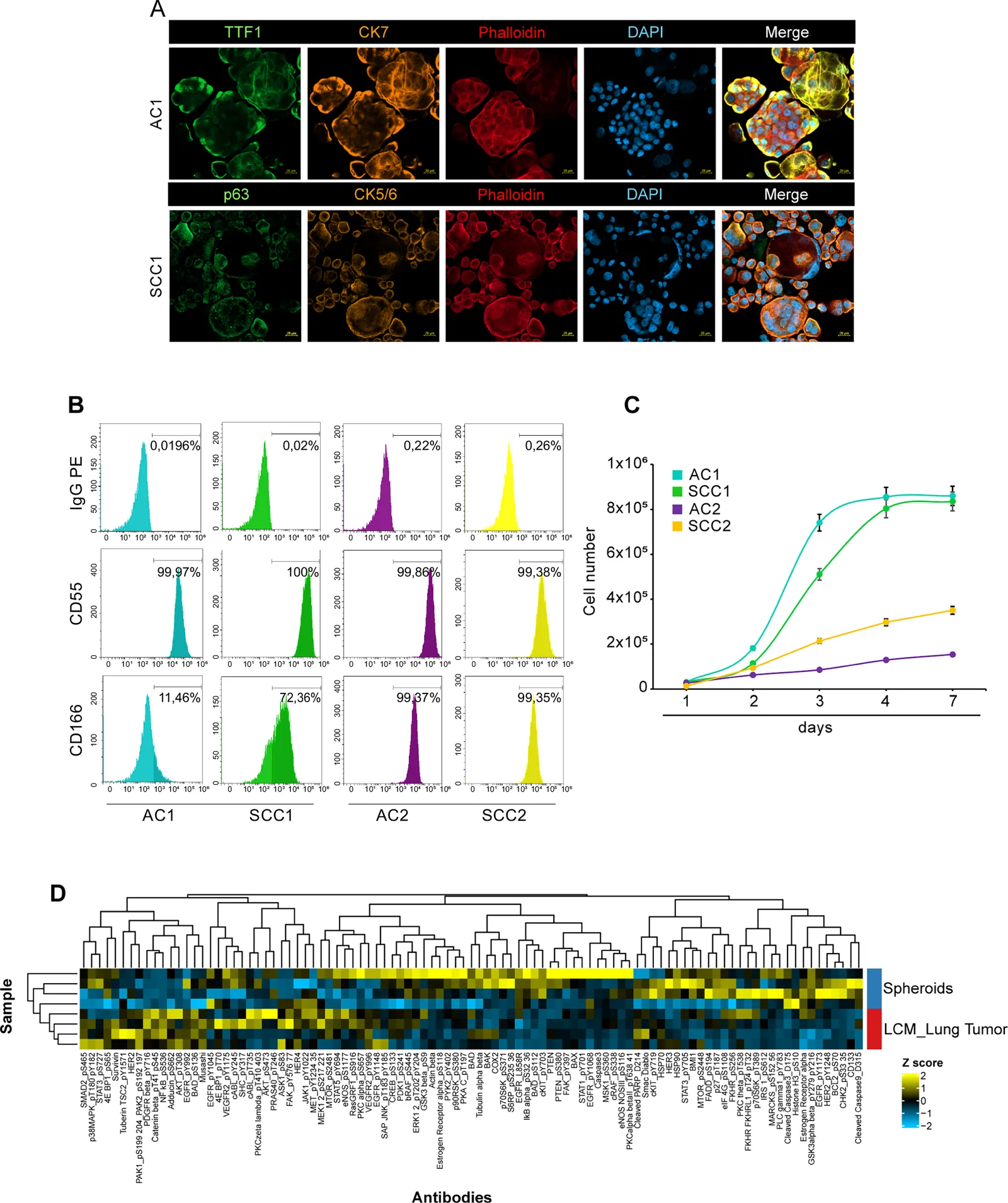
NSCLC spheroids characterization. **A** Immunofluorescence staining of AC (TTF1 and CK7) and SCC (p63 and CK5/6) markers in combination with phalloidin. Images were acquired at 20× magnification; scale bar: 20 μm. **B** Flow cytometry analysis of CD55 and CD166 expression in two SCC and two AC spheroid cultures **C** Proliferation kinetics of the spheroids used in (**B**). **D** Hierarchical clustering of RPPA data from four NSCLC patients obtained by laser-capture microdissection (LCM) of frozen tumor sections and their matched spheroids derived from distinct histotypes. The color-coded values (high = yellow, average = black, cyan = low) in the heatmap correspond to normalized intensities of averaged sample replicates (*n* = 3), standardized over the sample set analyzed (*z*-score). A list of RPPA antibodies used here is reported in Table S2.

Spheroids capture some essential features of tumor architecture as they contain spatially organized regions of hypoxia, senescence, proliferation, dormancy and differentiation [17, 18]. We used NSCLC surgical samples to generate spheroids that recapitulated the histology of parental tumors in immunocompromised mice [19–21]. NSCLC spheroids were cultured in serum-free medium in conditions promoting CSCs enrichment [20, 21], were regularly thawed to avoid culture-related changes, and periodically checked for in vivo tumorigenicity. Clinical features of NSCLC clinical samples are reported in Table S1. AC and SCC spheroids retained the phenotypic markers of the respective parental tumors (TTF1/CK7 for AC and p63/CK5/6 for SCC) (Fig. 1A). As a surrogate CSCs marker, we analyzed the expression of CD55, a molecule involved in lung cancer chemoresistance [22, 23] that was highly expressed in both AC and SCC spheroids (Fig. 1B). Also, we evaluated the expression of ALCAM/CD166, which is involved in NSCLC metastasis [24–26] and is overexpressed by slow proliferating NSCLC CSCs [27]. Accordingly, CD166 was particularly expressed in NSCLC spheroids with lower proliferation rates (Fig. 1B, C). To investigate intracellular signaling patterns, we performed Reverse-Phase Protein MicroArrays (RPPA) on NSCLC spheroids compared with respective parental tumors, the latter harvested through laser-capture microdissection of frozen surgical samples [28]. RPPA results showed that samples clustered according to their cell/tissue type (spheroids or tumors) (Fig. 1D). This result was initially surprising, as we expected that cells/tissues with the same genetic background would cluster together. However, at a closer look, these results indicate that microenvironmental conditions prevail over genetic features in dictating intracellular signaling, as has been recently observed in lung AC [29] and breast cancer [30]. In fact, relevant endpoints present in tumors or spheroids were indicative of cellular interactions with the respective microenvironment. In spheroids, we found an increased phosphorylation/activation of biosynthetic and proliferation-related endpoints (p70S6K_pT389 and p70S6K_pS371, GSK3alpha-beta_pY279-pY216 and GSK3-beta_pS9, Histone H3_pS10, cKIT_pY703 and cKIT_pY719, PLC-gamma1_pY783, EGFR_pY1173, FKHR_pS256 and FKHR-FKHRL1_pT24-pT32, bRAF_pS445), consistent with the presence of saturating growth factor concentrations. The spheroids cluster also showed an upregulation of the CSC marker CD133 [19] and of BMI1, involved in lung cancer progression [31, 32]. Moreover, spheroids showed increased levels of chemoresistance- (HSP90 and cRAF_pS338) and apoptosis-related factors (Cleaved Caspase3_D175, Cleaved Caspase9_D315, BAD, BAD_pS112), possibly due to the presence of dead cells at the center of larger spheroids. Differently, the tumor cluster was characterized by cytokine-mediated signaling and inflammation (NF-kB_pS536, AKT_pT308, STAT3_pS727, SMAD2_pS465, p38MAPK_pT180-pY182, Catenin beta_pT41 pS45, PDGFR-beta_pY716, EGFR_pY992). Tumors also upregulated cytoskeleton-related factors such as Adducin_pS662 and p21-activated kinase (PAK1_pS199-204, PAK2_pS192-197), the latter known to promote angiogenesis (VEGFR2_pY996), metastasis, and cell survival [33]. Altogether, these observations show that NSCLC spheroids maintain the phenotype of original tumors (AC or SCC), are enriched in CSCs, and are highly responsive to microenvironmental conditions, representing a suitable model to investigate tumor crosstalk with immune cells.

### Characterization of cell–cell interactions in co-cultures of NSCLC spheroids and macrophages

**Fig. 2:**
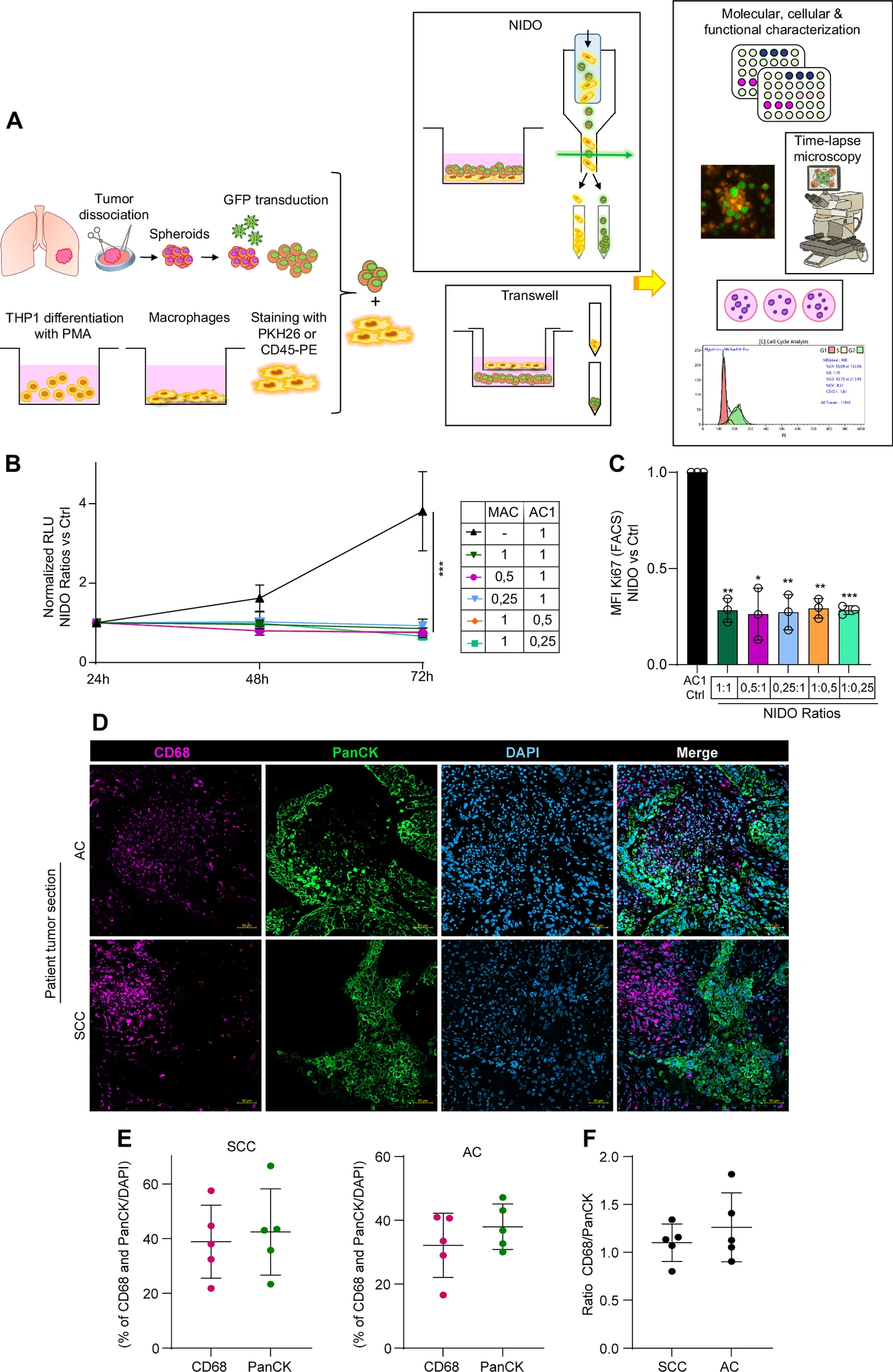
Schematic workflow of NIDO cultures generation, characterization and determination of macrophage/tumor cell ratio. **A** Spheroids were directly generated from surgically resected NSCLC specimens obtained from two adenocarcinoma (AC) and two squamous cell carcinoma (SCC) patients. To generate spheroid-macrophage co-cultures (NIDO cultures), AC and SCC spheroids were expanded and transduced with a GFP-encoding lentiviral vector. In parallel, THP-1 cells were differentiated into macrophages with PMA and subsequently co-cultured with spheroids, after staining with PKH26 or CD45-PE. NIDO cultures were then analyzed for their morphological, molecular and functional properties. **B** Left panel: cell proliferation curve based on ATP detection (CellTiter-GLO^TM^) of AC1 spheroids co-cultured with THP-1-derived macrophages at different ratios, as reported in the corresponding table (right panel). Data are the mean ± SD of three independent experiments. **C** Mean Fluorescence Intensity (MFI) values of Ki67 expression assessed by flow cytometry in AC1 spheroids upon NIDO culture with THP-1 macrophages at different ratios. The histograms show the normalized values of Ki67 MFI in NIDO samples as compared to controls (AC1 spheroids alone). The corresponding plots are shown in Fig. S1. Data are the mean ± SD of three independent experiments. **D** Representative confocal images of FFPE sections obtained from patients with lung adenocarcinoma (AC) and squamous cell carcinoma (SCC). Sections were stained for the macrophage marker CD68 (purple) and for pan-cytokeratin (PanCK, green) for NSCLC cells. Nuclei were counterstained with DAPI (blue). **E** Quantitative analysis of CD68- and PanCK-positive areas, normalized to DAPI-positive areas and expressed as percentage values, performed using the ImageJ software. Data were obtained from 5 random fields per section. **F** Ratio between CD68 and PanCK values is shown in Fig. 2E. Statistical significance, where applicable, was determined by comparing each experimental condition with the control and with the other experimental groups: ***P < 0.001 (one-tailed t-test and n.s. = not significant).

**Fig. 3:**
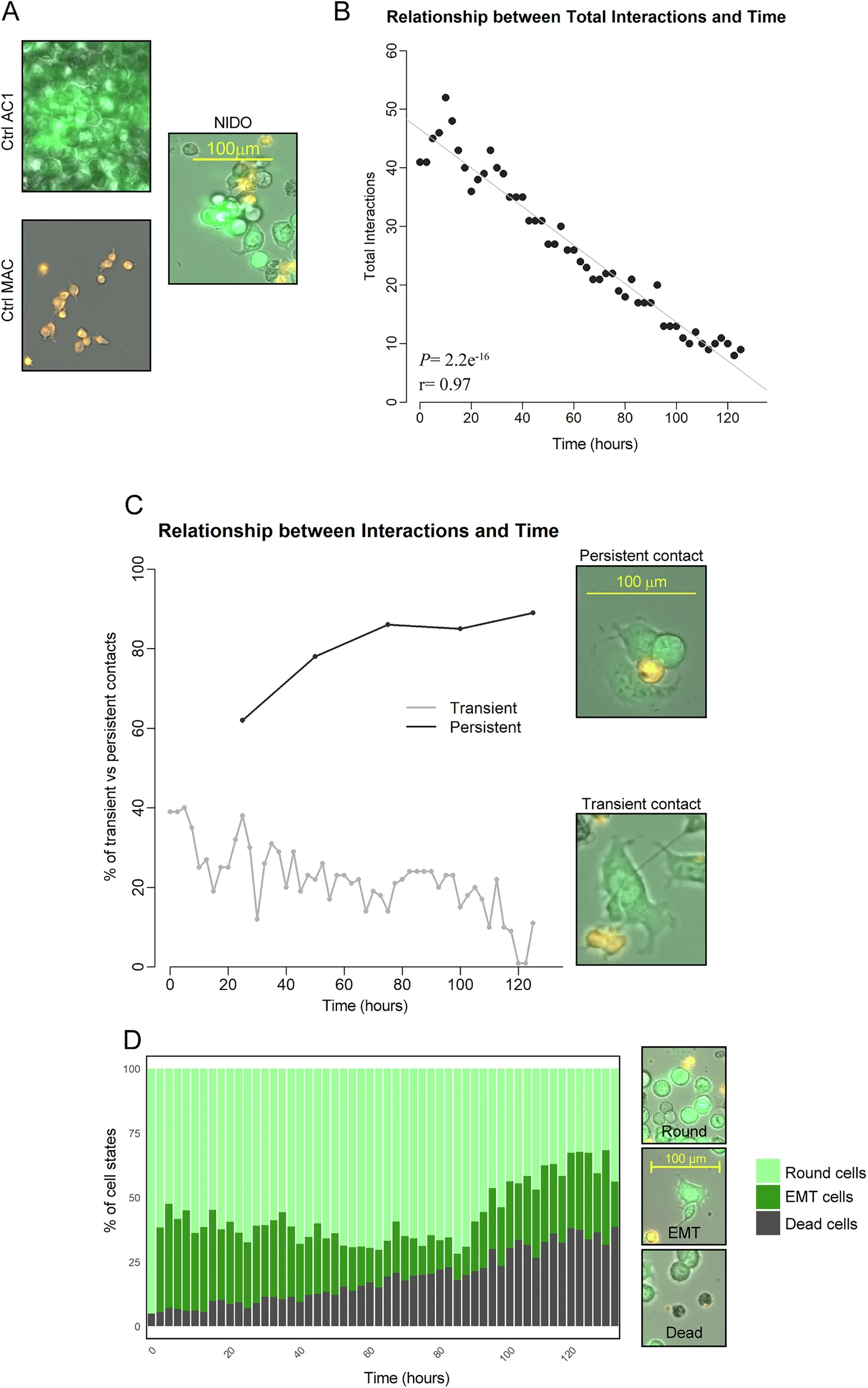
Time-lapse imaging analysis of NIDO cultures dynamics. **A** Time-lapse phase contrast/fluorescence images of AC1 spheroid cultures (Ctrl AC1, green), macrophages (Ctrl MAC, orange), or both cell types interacting in NIDO cultures (NIDO). Scale bar: 100 μm. **B** Total interactions in NIDO cultures over time. The Y axis indicates the total number of interactions, while the X axis represents the corresponding time points. The gray line corresponds to the fitted linear regression, showing a strong negative correlation (r = 0.97, *P* = 2.2e^–16^) and a progressive linear decrease of interactions throughout the observation period. **C** Dynamics of interactions in NIDO cultures divided according to the type of cell–cell contact (transient or persistent) with representative images (right panels) taken from Video S1. The Y axis shows the relative percentage of transient versus persistent contacts while the X axis represents the corresponding time points. Transient interactions were defined as cell–cell interactions lasting ≤2.5 h, whereas persistent interactions were defined as cell–cell interactions lasting ≥25 h. Values were obtained by manually counting one frame containing approximately 150-270 cells every 2 h (small gray spots). **D** Percentage of cell states in NIDO cultures, defined as round cells (light green bar), EMT cells (dark green bar), and dead cells (black bar) with representative images (right panels) taken from Video S1.

To characterize NSCLC cells-macrophages interactions, we used co-cultures of spheroids and THP-1-derived macrophages, the latter being widely used as an alternative to primary human macrophages [34]. A workflow of the study is shown in Fig. 2A. Briefly, NSCLC spheroids were transduced with a vector encoding the green fluorescent protein (GFP), while THP-1 cells were differentiated into macrophages with phorbol myristate acetate (PMA) and stained with the orange-emitting dye PKH26 or with CD45-PE. Then, the two cell types were co-cultured for 1–5 days, depending on the experimental endpoint. Co-cultures were named NIDO, an acronym for Niche-Induced Dormancy. Also, NIDO is the Italian word for “nest”, due to the presence of nest-shaped cellular aggregates (picture in Fig. 2A). To choose an effective ratio between macrophages and tumor cells, we co-cultured different ratios of the two cell types and analyzed the effects in terms of tumor cells’ proliferation and Ki67 expression. Figure 2B shows the rates of AC proliferation in spheroid cultures alone or in NIDO cultures produced with different ratios of macrophages. Surprisingly, we found that all the macrophage concentrations tested induced an abrupt proliferative arrest of tumor cells in NIDO cultures (Fig. 2B). According to this observation, Ki67 levels (expressed as mean fluorescence intensity, MFI) dropped in the presence of macrophages at every ratio tested (Fig. 2C). Notably, the overall percentage of Ki67-positive AC cells did not undergo major changes between control cultures (98.3%) and NIDO cultures (ranging from 96.2 to 94.3%). By contrast, Ki67 MFI varied substantially between control cultures (MFI: 23575) and all the NIDO conditions (MFI ranging from 8041 to 9409) (Fig. S1). These observations are in line with previous studies showing that, when cells progress into quiescence, Ki67 expression is regulated in a graded rather than on/off fashion [35]. Then, we investigated which macrophage: tumor cells ratio was actually present in the NSCLC TME. Thus, we assessed macrophage: tumor cells ratios in tumor sections obtained from two NSCLC patients (AC and SCC) stained with anti-CD68 and anti-pan-cytokeratin antibodies to detect macrophages and tumor cells, respectively (Fig. 2D). Macrophage: tumor cells ratios were in the range of 0.9-1.8 for AC and of 0.8-1.3 for SCC (Fig. 2E, F), thus supporting the use of a 1:1 macrophage: tumor cells ratio in NIDO cultures as a fair approximation of the ratio actually occurring in the TME. To further characterize heterotypic cell–cell interactions in NIDO cultures, these were (i) examined by time-lapse microscopy to assess cell morphology, dynamism and viability, and (ii) subjected to fluorescence-activated cell sorting (FACS) to analyze changes in the proliferation, stemness, EMT and chemoresistance of NSCLC cells. First, we investigated the quality, frequency and phenotypic effect of interactions between NSCLC cells and macrophages through time-lapse microscopy. NIDO cultures were monitored for 5 days (Video S1). Figure 3A shows AC cells (upper panel, green) and macrophages (lower panel, orange) before (left) and during NIDO culture (right). Figure 3B reports heterotypic cell–cell interactions over time, showing these were more dynamic in the first 24 h and then gradually decreased. We noticed two main types of interactions, defined as transient (lasting ≤2.5 h) or persistent ( ≥ 25 h). Figure 3C shows that transient interactions gradually decreased over time, although not uniformly, whereas persistent interactions had the opposite trend. Then, we analyzed tumor cell phenotype over time, distinguishing between round-shaped cells, flat spindle-like cells (EMT cells), and dead cells. As shown in Fig. 3D, control spheroid cultures consisted entirely of round cells with a small physiological fraction of dead cells. Co-culture with macrophages immediately induced the appearance of 10–40% EMT cells that persisted throughout the experiment. The percentage of dead cells increased constantly over time, peaking around 38% after 5 days of co-culture. The parallel increase of both persistent cell–cell interactions and cell death suggests that some prolonged interactions between tumor cells and macrophages result in tumor cell killing. However, cell–cell interactions also induced molecular and functional changes that were explored in subsequent experiments. Additionally, we analyzed macrophage modifications induced by NIDO cultures in terms of M1/M2 polarization (Fig. S2). Macrophages derived from NIDO cultures with AC or SCC spheroids were analyzed for M1 and M2 markers as compared to M0 and to positive controls. The latter were obtained by incubating PMA-treated macrophages (corresponding to the M0 state) with cytokines inducing M1 (lipopolysaccharide, LPS) or M2 (IL-4 and IL-13) polarization (Fig S2A, B). NIDO cultures induced an increase in M2 markers MRC1 and IL-10, but also in the M1 marker TNFα (Fig. S2C, D). These observations suggest a prevalent M2 polarization of NIDO macrophages but are also consistent with TAMs’ complex phenotype.

### Physical contact with macrophages induces dormancy of lung cancer cells

**Fig. 4:**
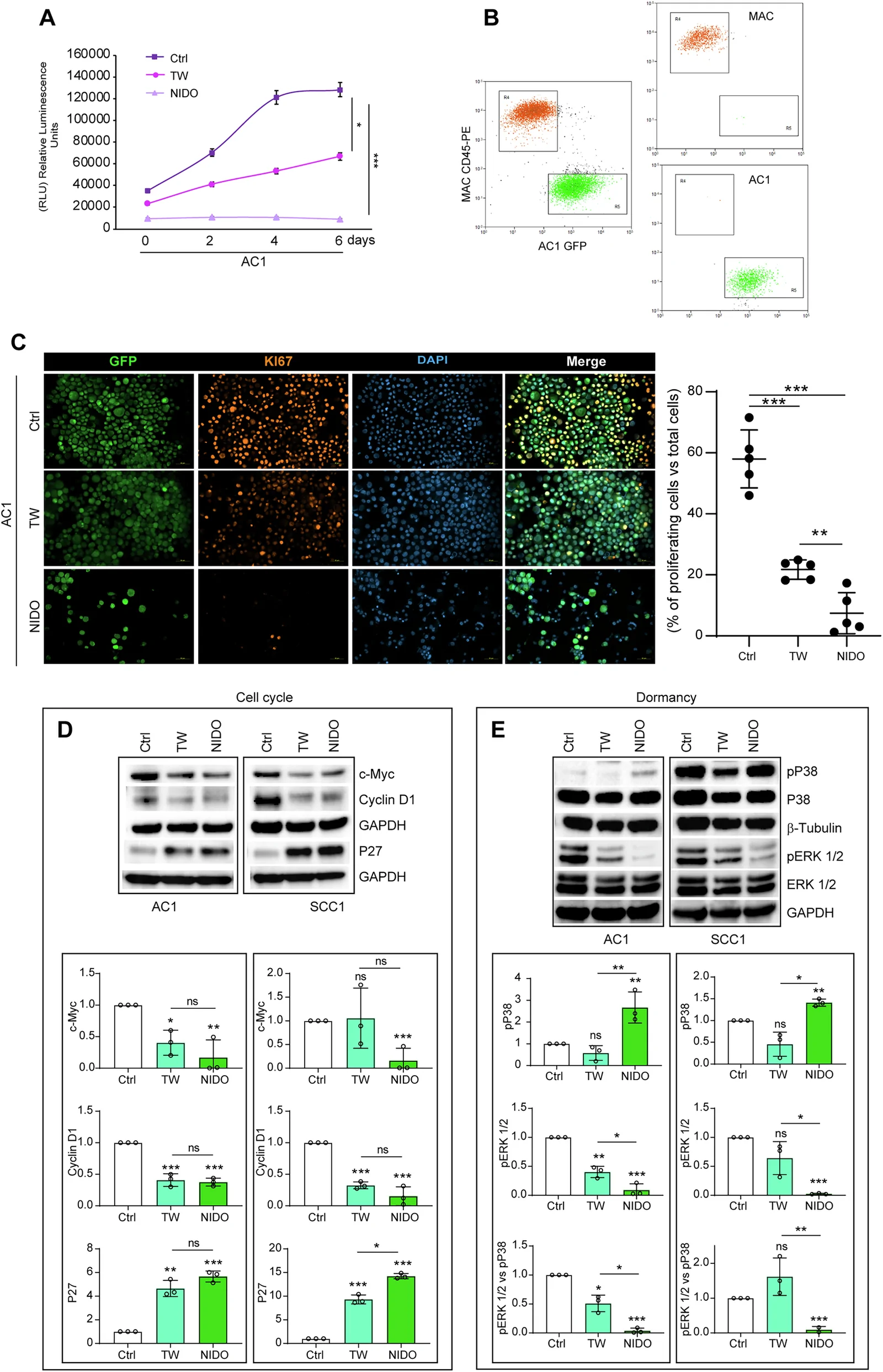
NIDO cultures induce a dormant state in NSCLC cells. **A** Results of a proliferation assay (CellTiter-GLO^TM^) reported as Relative Luminescent Units (RLU) at different time points of control AC1 (dark violet square), transwell-derived AC1 (TW, dark pink circle), and NIDO-derived AC1 (light violet triangle). Data are the mean ± SD of three independent experiments. **B** FACS-based separation of CD45-positive macrophages (MAC) and GFP-positive spheroid populations (AC1) upon NIDO cultures (left) and post-sorting control of population cell purity (right). **C** Left: Representative confocal images of GFP-positive AC1 spheroids, stained with the proliferation marker Ki67 (Orange) and DAPI for nuclei (blue), derived from control (Ctrl), transwell (TW), and NIDO cultures. Right: Quantitative analysis of Ki67-positive cells normalized to GFP/DAPI-positive cells (area) and reported as percentages was conducted using ImageJ software. Data were obtained from three independent sets, each consisting of five fields per group. **D, E** Upper panels: immunoblot analysis of cell cycle (c-Myc, Cyclin D1 and P27) and dormancy (pP38, total P38, pERK 1/2, ERK 1/2) factors on whole lysates of AC1 and SCC1 spheroids derived from control (Ctrl), transwell (TW) and NIDO cultures. GAPDH and β-Tubulin were used as loading controls. Images are representative of immunoblot experiments shown in the Supplementary file 1 original western blot. Bottom panels: quantification of immunoblot experiments, where data are expressed as Mean ± SD *n* = 3. Statistical significance was determined by comparing each experimental condition with the control and with the other experimental groups: *P < 0.05, **P < 0.01 and ***P < 0.001 (one-tailed t-test, n.s. = not significant).

To investigate the functional effects induced by macrophages on NSCLC cells, we compared tumor cell proliferation in two conditions: transwell cultures, where tumor cells and macrophages are separated by a porous membrane allowing the exchange of soluble factors, and NIDO cultures, where cells are in physical contact. We observed that transwell cultures induced a proliferative slowdown, whereas NIDO cultures resulted in a complete proliferative arrest of tumor cells (Figs. 4A and S3A). Cell cycle analysis of NSCLC cells in control, transwell, or NIDO cultures (the latter FACS-sorted) (Fig. 4B) showed that G0/G1 cells increased in transwell cultures and further in NIDO cultures (Fig. S3B, C). Immunofluorescence (IF) analysis of the proliferation marker Ki67 showed a decrease in Ki67-positive nuclei in transwell-cultured NSCLC cells and an almost complete disappearance in NIDO cultures (Figs. 4C and S3D). Immunoblot analysis showed a decrease of factors involved in cell cycle progression such as c-Myc, cyclin D1 and a parallel increase of cyclin-dependent kinase inhibitor 1B (p27Kip1) in both transwell and NIDO cultures (Fig. 4D). However, analysis of the phospho-P38 (pP38)/phospho-ERK1/2 (pERK) ratio, which is recognized as a hallmark of cancer dormancy [36], showed a strong decrease of pERK/pP38 in NIDO but not in transwell cultures, showing that NSCLC cells undergo dormancy upon physical contact with macrophages (Fig. 4E).

### Co-culture with macrophages stimulates stemness, chemoresistance and EMT of NSCLC CSCs

**Fig. 5:**
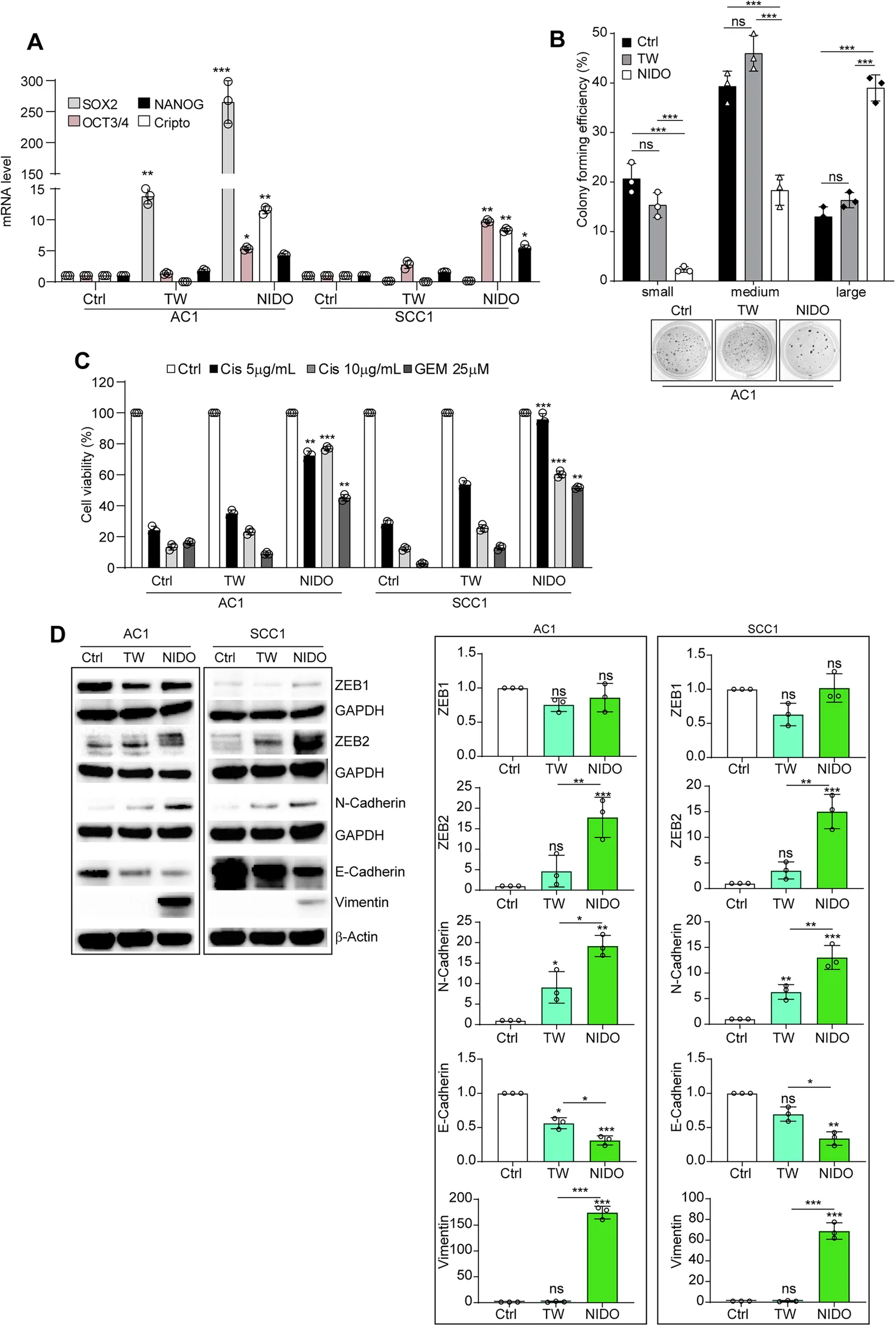
NIDO cultures promote stemness and EMT traits in NSCLC cells. **A** qRT-PCR of the indicated transcripts in control (Ctrl), transwell (TW) and NIDO-cultured AC1 (left) and SCC1 (right) spheroids. Data are the mean ± SD of three technical replicates. **B** Upper panel: colony-forming assay performed on control (Ctrl), transwell (TW) and NIDO-derived AC1 spheroids. The results are evaluated as colony formation in semisolid culture after 4 weeks and expressed as normalized colony size/percentage over plated cells. Bottom panel: representative pictures of soft agar plates. Data are the mean ± SD of three independent experiments. **C** Viability of AC1 (left) and SCC1 (right) spheroids derived from control (Ctrl), transwell (TW), and NIDO cultures treated for 48 h with 5-10 μg/mL cisplatin (Cis) or 25 μM gemcitabine (GEM). Data are the mean ± SD of three technical replicates. **D** Left panels: immunoblot analysis of EMT factors (ZEB1, ZEB2, N-Cadherin, E-Cadherin, Vimentin) on whole lysates of AC1 and SCC1 spheroids derived from control (Ctrl), transwell (TW), and NIDO samples. GAPDH and β-Actin were used as loading controls. Right panels: quantification of immunoblot experiments, where data are expressed as Mean ± SD *n* = 3. Images are representative of immunoblot experiments shown in Supplementary file 1 original western blot. Statistical significance was determined by comparing each experimental condition with the control and with the other experimental groups: *P < 0.05, **P < 0.01 and ***P < 0.001 (one-tailed t-test, n.s. = not significant).

Tumor dormancy is often associated with stemness, chemoresistance and EMT in tumor cells [37]. To assess whether macrophages induced such state transitions in NSCLC cells, we evaluated the expression of stemness-associated factors SOX2, OCT3/4 and NANOG by real-time PCR in transwell or NIDO cultures. We also assessed Cripto expression, as this factor is involved in NSCLC stemness and chemoresistance [38]. In both AC and SCC, we observed a significantly higher expression of stemness-related factors upon NIDO cultures as compared to control and transwell cultures (Fig. 5A). In line with these observations, colony-formation assays showed that tumor cells previously grown in NIDO cultures generated a significantly higher number of larger colonies as compared to control and transwell cultures (Fig. 5B), indicating an increased repopulating capacity. Then, we investigated the sensitivity of AC and SCC cells to chemotherapeutic agents used for NSCLC, i.e., cisplatin and gemcitabine. Despite minor differences between AC and SCC, cells in NIDO cultures showed higher chemoresistance as compared to control and transwell cultures (Fig. 5C). Notably, the viability of NIDO-derived SCC at lower cisplatin concentrations was approximately 100%, versus 25% of controls and 50% of transwell cultures, indicating that macrophages activate strong chemoresistance programs in NSCLC. Finally, we evaluated EMT factors by immunoblotting. We observed an increase of N-cadherin and vimentin, and a parallel decrease of E-cadherin, collectively indicative of EMT in NIDO-cultured NSCLC cells, while the same effects were weaker or absent in transwell-cultured cells (Fig. 5D). ZEB1 and ZEB2 showed a different trend, with only ZEB2 increasing upon NIDO cultures, in line with its reported role in quiescence and/or chemoresistance in colorectal and other tumors [39–42]. Overall, these findings support the hypothesis that macrophages promote stemness, EMT and chemoresistance in NSCLC with a prevalent role played by cell–cell interactions.

### Reciprocal modulation of TGF-β in NIDO-cultured cells

**Fig. 6:**
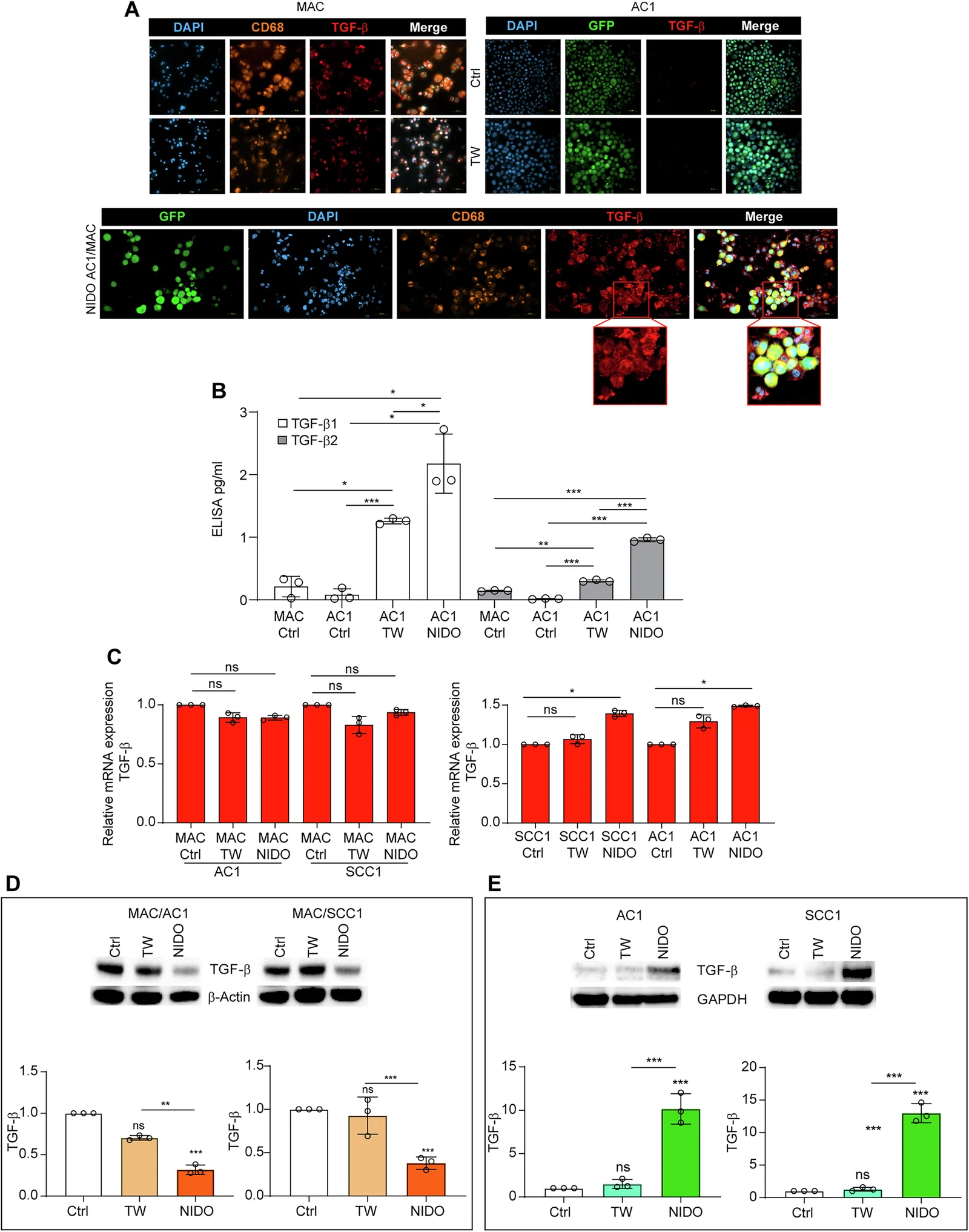
TGF-β expression in macrophages and AC1 spheroids upon NIDO cultures. **A** Upper panels: Representative confocal images of macrophages (MAC) and AC1 cells (AC1) cultured alone (Ctrl) or in transwell devices (TW). Lower panels: Representative confocal images of NIDO cultures. Macrophages (MAC) were stained for CD68 (orange) and TGF-β (red). GFP-positive (green) AC1 spheroids were stained for TGF-β (red). Nuclei of both cell types were stained with DAPI (blue) (20x magnification, scale bar 20 μm). **B** Analysis of secreted TGF-β1 and TGF-β2 levels detected by ELISA under different experimental conditions: macrophages (MAC) and AC1 spheroids (AC1) in control cultures (Ctrl), transwell (TW) and NIDO cultures. Data are the mean ± SD of three technical replicates. **C** qRT-PCR analysis of TGF-β mRNA levels in MAC (left), AC1 and SCC1 spheroids (right) in control (Ctrl), transwell (TW), and NIDO cultures. Data are the mean ± SD of three independent experiments. **D, E** Upper panels: Western blot analysis of intracellular TGF-β expression in macrophages and NSCLC cells, cultured alone (Ctrl), in transwell (TW) or NIDO conditions. GAPDH was used as a loading control. Bottom panels: quantification of immunoblot experiments, where data were presented as Mean ± SD *n* = 3. Images are representative of immunoblot experiments shown in Supplementary file 1 original Western blot. Statistical significance was determined by comparing each experimental condition with the control and with the other experimental groups: *P < 0.05, **P < 0.01 and ***P < 0.001 (one-tailed t-test, n.s. = not significant).

Transforming Growth Factor-β (TGF-β) family proteins play a key role in tumor cell dormancy [43]. In the lungs, TGF-β2 produced by alveolar macrophages has been recently shown to induce dormancy of disseminated breast cancer cells [6]. To assess whether TGF-β treatment would reproduce macrophage effects on NSCLC cells, we treated AC and SCC cells with purified recombinant TGF-β, and we assessed proliferation, expression of EMT- and stemness-related markers, chemosensitivity and colony-forming ability. TGF-β was able to inhibit spheroid proliferation (Fig. S4A), to increase the expression of OCT3/4, SOX2, Cripto, NANOG and EMT-related factors (vimentin, E-cadherin, SNAI1, SNAI2, ZEB1 and ZEB2) (Fig. S4B, C), to induce chemoresistance (Fig. S4D) and to support colony-forming capacity even in the presence of cisplatin (Fig. S4E). Based on these premises, we hypothesized that TGF-β could promote macrophage-mediated effects in co-cultures. Therefore, we analyzed TGF-β expression by IF in either macrophages (stained for CD68, orange) or NSCLC cells (GFP-positive, green). As expected, macrophages, but not NSCLC cells, had a high basal expression of TGF-β, and this remained unchanged in transwell cultures, although the protein acquired a polarized pattern (Figs. 6A and S5A). In NIDO cultures, TGF-β was abundantly expressed, but its expression was not confined to macrophages, being localized also in tumor cells (Fig. 6A, magnification). Measurement of secreted TGF-β1 and TGF-β2 by ELISA also confirmed the presence of both proteins in the supernatant of macrophages (but not of tumor cells alone) and a progressive increase in transwell and NIDO cultures, suggesting that both TGF-β1 and TGF-β2 may be involved in macrophages-tumor crosstalk (Figs. 6B and S5B). In order to determine the exact localization of TGF-β in co-cultured cells, we analyzed TGF-β expression at the RNA and protein level in macrophages and tumor cells upon NIDO culture and subsequent FACS separation. TGF-β RNA levels upon co-culture remained unchanged in macrophages and showed a small but significant increase in tumor cells (Fig. 6C). Then, we analyzed the levels of intracellular TGF-β in tumor cells and in macrophages. Unexpectedly, we found that TGF-β underwent a specular decrease in macrophages and an increase in tumor cells upon NIDO culture but not upon transwell culture (Fig. 6D, E). The simultaneous loss of TGF-β from macrophages and acquisition by tumor cells in the absence of RNA modulation led us to hypothesize that TGF-β could be transferred from macrophages to neighboring tumor cells, according to a reported local exchange of TGF-β within cell–cell circuits [44].

### Inhibition of autophagosome-mediated TGF-β secretion abolishes co-culture effects on NSCLC cells

**Fig. 7:**
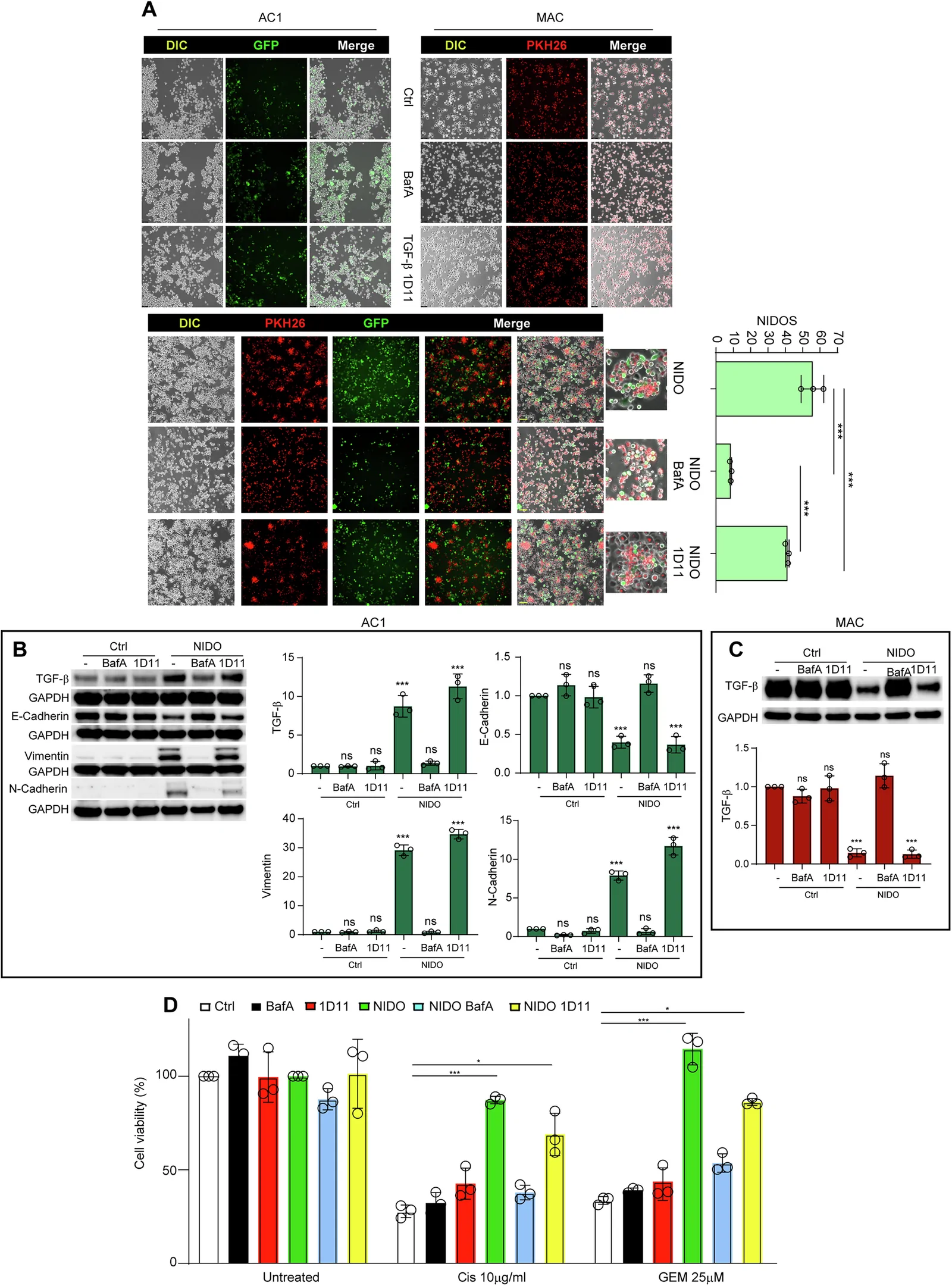
Effects of TGF-β inhibition in NIDO cultures. **A** Phase-contrast (differential interference contrast, DIC) and fluorescent microscopy images showing GFP-positive (green) AC1 spheroids (AC1) and PKH26-positive (red) macrophages (MAC) under different experimental conditions. Upper panels show AC1 or MAC cultured alone (Ctrl), whereas lower left panels show NIDO cultures untreated or treated with BafA (6 nM) or with the neutralizing anti-TGF-β antibody 1D11 (4,62 μg/mL) for 48 h (10× magnification; scale bar: 100 μm). The bottom right panel shows a quantification of the presence of heterotypic cellular nests (NIDOS) in co-cultures treated with BafA (6 nM) or 1D11 (4,62 μg/mL). Data were obtained from three independent sets, each consisting of three fields per group. **B** Western blot analysis of TGF-β levels and EMT-related proteins (E-Cadherin, Vimentin, N-Cadherin) in AC1 spheroids in control (Ctrl) and NIDO cultures treated with BafA (6 nM) or 1D11 (4,62 μg/mL. GAPDH was used as a loading control. Densitometric quantification is shown on the right. **C** Western blot analysis of TGF-β levels in MAC, in control (Ctrl) and NIDO cultures treated with BafA (6 nM) or 1D11 (4,62 μg/mL). GAPDH was used as a loading control. Densitometric quantification is shown in the bottom panel; data were presented as Mean ± SD *n* = 3. Images in (**B, C**) are representative of immunoblot experiments shown in the Supplementary file 1 original Western blot. **D** Cell viability assay (CellTiter-Glo^TM^) performed on control (Ctrl) or NIDO-derived AC1 spheroids left untreated, pre-treated with BafA (6 nM) or with 1D11 (4,62 μg/mL) and then treated with chemotherapeutic agents cisplatin (Cis 10μg/mL) or gemcitabine (GEM 25 μM) for 48 h. The graph shows the percentage of viable cells. Data are the mean ± SD of three technical replicates. Statistical significance was determined by comparing each experimental condition with the control and with the other experimental groups: *P < 0.05 and ***P < 0.001 (one-tailed t-test, n.s. = not significant).

Due to its crucial activities in embryo development, immune regulation and cancer, TGF-β production, secretion and activation are tightly regulated [45]. To understand the mechanism(s) underlying TGF-β regulation in NIDO cultures, we compared two approaches. First, we treated NIDO cultures with 1D11, a humanized monoclonal antibody (MoAb) that neutralizes extracellular TGF-β1, TGF-β2 and TGF-β3, preventing activation of downstream pathways [46, 47]. Secondly, we treated NIDO cultures with Bafilomycin A1 (BafA), which interferes with TGF-β secretion by macrophages through the autophagic route [48]. Specifically, BafA inhibits the vacuolar-type H^+^-translocating ATPase, blocking autophagosome-lysosome fusion and disrupting the autophagic flux [49]. Also, BafA can indirectly affect cell–cell contact by interfering with the trafficking of adhesion molecules [50]. First, we titrated BafA and MoAb 1D11 concentrations on macrophages and NSCLC spheroids accurately to avoid toxic effects (Fig. S6). Then, we treated NIDO cultures with BafA or MoAb 1D11, and we evaluated the effects on cell morphology and interaction. Tumor cells in BafA-treated NIDO cultures were smaller and round-shaped (Fig. 7A). Treatment with BafA abrogated cell–cell contact and reduced by 90% the number of nests, whereas MoAb 1D11 only partially inhibited nest formation (Fig. 7A). Then, we analyzed TGF-β levels in NSCLC spheroids and macrophages. Neither BafA nor MoAb 1D11 influenced TGF-β levels in control cultures. However, BafA, but not MoAb 1D11, abrogated the decrease of TGF-β in macrophages and the parallel increase in NSCLC cells (Fig. 7B, C), indicating that this phenomenon depends on autophagosome-mediated trafficking. Moreover, BafA counteracted EMT marker modulation in NSCLC spheroids, indicating that EMT was also dependent on functional autophagic vesicles (Fig. 7B). Finally, we analyzed the effect of BafA or MoAb 1D11 on NSCLC cells’ chemoresistance. Notably, BafA largely abrogated both cisplatin and gemcitabine resistance induced by NIDO culture (Fig. 7D). Inhibition of soluble TGF-β by 1D11 had a weaker but still significant effect, suggesting that extracellular TGF-β, possibly produced by tumor cells in an autocrine fashion, contributes to NSCLC cells’ chemoresistance (Fig. 7D). Based on these observations, we propose a model in which macrophage-produced TGF-β is transferred to neighboring tumor cells through autophagosome-mediated trafficking/secretory autophagy. TGF-β uptake then promotes CSCs plasticity, resulting in increased tumor fitness. In control cultures of macrophages or spheroids, TGF-β did not accumulate in the presence of BafA, suggesting that, in the absence of heterotypic cell–cell interactions, TGF-β secretion occurs through the classical endoplasmic reticulum-Golgi pathway. The unconventional pathway relying on secretory autophagy [48] seems to occur prevalently in co-culture conditions, suggesting a yet unexplored route leading to macrophage-induced NSCLC chemoresistance.

## Discussion

The interaction of lung tumors with the immune microenvironment crucially affects treatment choice and prognostic performance. Within the immune TME, macrophages play a prominent role in the formation of a pro-tumorigenic niche for NSCLC cells [5, 51], thus representing candidates for immunomodulatory strategies [52]. By using co-cultures of NSCLC patient-derived spheroids with THP-1-derived macrophages, we monitored tumor cells-macrophages interactions accurately. We demonstrated that physical interactions between macrophages and tumor cells are essential to produce a full range of state transitions, including dormancy, EMT, stemness, and chemoresistance. Then, we identified TGF-β as a key factor involved in macrophages-NSCLC crosstalk. In fact, we observed that TGF-β was released by macrophages through an autophagosome-dependent mechanism, promoting heterotypic cellular aggregation and tumor cell plasticity. Our work provides some important elements for further reflection. First, macrophages can establish persistent interactions with tumor cells, thus creating specialized pro-tumorigenic niches. Similar structures have also been reported by other investigators: Dalla and coworkers showed that disseminated breast cancer cells closely interact with macrophages, activating mesenchymal and growth arrest programs [6]. In early-stage lung cancer, macrophages accumulate close to tumour cells and induce pro-metastatic programs [5]. La Fleur et al. identified a distinct subpopulation of TAMs expressing the scavenger receptor MARCO that aggregated in close proximity to tumor cell nests [53]. Macrophagic niches represent a specialized microenvironment for NSCLC cells, where the two cell types establish cell–cell circuits with reciprocal advantages [54]. Cell–cell communication within macrophagic niches relies on contact-dependent mechanisms but also on the formation of a local microenvironment enriched in growth factors and cytokines [54]. Interestingly, both in the nervous system and in the skin, TGF-β has been shown to be released and activated locally, acting in a spatially confined cellular milieu rather than in a diffusible fashion [44, 55]. These findings are consistent with our observation that TGF-β is released from macrophages and accumulates in neighboring tumor cells where it promotes transition to a stem-like chemoresistant phenotype. Interestingly, we observed that in NIDO cultures TGF-β is secreted through an unconventional pathway inhibited by BaFA and dependent on the autophagic machinery [48]. TGF-β-secretion through the autophagosome pathway apparently required a specialized milieu formed by the close interaction between the two cell types, as it was not present in transwell cultures. It is also conceivable that the local release/activation of TGF-β results in a maximal effect on tumor cells. In fact, we observed that the diffuse presence of TGF-β in the culture medium (either in transwell cultures or upon exogenous administration) results in a proliferative slowdown and mild effects on EMT/chemoresistance, whereas NIDO cultures induced a complete proliferative arrest and strong EMT/chemoresistance. Besides this observation, it is also likely that in NIDO cultures the crosstalk between macrophages and neighboring NSCLC cells may activate other pathways besides TGF-β, such as WNT and HIPPO, contributing to CSC plasticity [56]. Notably, tumor cell dormancy in NIDO cultures is accompanied by an increase in stemness and chemoresistance. The association between dormancy, stemness, and drug resistance has been observed in several tumors, indicating dormancy as a high-potential state where tumor cells accumulate both genetic and epigenetic changes useful for their survival and adaptation [37]. Interestingly, tumor cell dormancy in NIDO cultures was accompanied by ZEB2 overexpression, according to our previous finding that increased ZEB2 expression is found in quiescent lung cancer cells [27]. Additionally, ZEB2 has been shown to be functionally related to dormancy and chemoresistance in colorectal, breast, and endometrial tumors [39–42]. Also, according to these observations, ZEB2 upregulation in NSCLC cells has been recently reported to induce TAM polarization towards a TKI-resistant state [57], suggesting that ZEB2 may be involved in inducing both chemo- and immunotherapy resistance in NSCLC cells. Our findings provide new insights into the interactions between NSCLC cells and macrophages, showing the existence of a cell–cell circuit mediated by TGF-β and able to promote CSC plasticity. These observations contribute to explaining how macrophages influence tumor progression and chemoresistance, and may provide new insights for future strategies aimed at macrophage targeting in NSCLC.

## Materials and methods

### Antibodies and reagents

Thyroid Transcription Factor 1 (TTF-1) (D2E8) Rabbit #12373, p63 (D9L7L) XP® Rabbit #39692, c-Myc (D84C12) Rabbit #5605, ZEB1 (D80D3) Rabbit #3396, N-Cadherin (D4R1H) XP® Rabbit #13116, E-Cadherin (24E10) Rabbit #3195, Vimentin (D21H3) XP® Rabbit #5741, TGF-β Rabbit #3711, p-p38 MAPK Mouse (E-1) # sc-166182, p38 MAPK Rabbit #9212 were from Cell Signaling Technology (Danvers, MA, USA). Cyclin D1 (A-12) Mouse #sc-8396, P27 (F-8) Mouse # sc-1641, and CD68 (3F103) Mouse # sc-70761 were from Santa Cruz Biotechnology (Dallas, TX, USA). ZEB2 Rabbit # SAB3500515 was from Thermo Fisher Scientific Inc. (Waltham, MA, USA). GAPDH Rabbit # G9545, anti-β-Tubulin Mouse # T5293, anti-β-Actin (ACTB) #A5441 were from Merck (Rahway, NJ, USA). TGF-β1 Rabbit #NBP1-80289 (Novus Biologicals, Centennial, CO, USA). CK7 (OV-TL 12/30) Mouse #M701801-2, Ki67 (MIB-1) Mouse #M7240, Pan Cytokeratin Rabbit # Z0622 were from Dako Agilent Technologies, Inc. (Santa Clara, CA, USA). CD45-PE (5-B1) Mouse #130-080-201, Cytokeratin 5/6-PE (REA913 D5/16), REAfinity™ # 130-115-257, Miltenyi Biotec (Bergisch Gladbach, Germany). Phalloidin Alexa Fluor™ 647 # A22287, donkey anti-rabbit IgG Alexa Fluor™ 488 #A-21206, donkey anti-mouse Alexa Fluor™ 555 # A-31570, DAPI #62248, Phorbol 12-myristate 13-acetate, 95% (PMA) # J63916. Rabbit IgG (H + L) Secondary Antibody #31460, Rabbit IgG (H + L) Secondary Antibody #31460 were from Thermo Fisher Scientific Inc. Anti-Human CD55-PE Mouse (IA10 (RUO) #555694, PE Mouse IgG2a, κ Isotype Control (G155-178 (RUO)) #553457, Anti-Human CD166-PE Mouse (3A6 (RUO)) #559263, PE Mouse IgG1, κ Isotype Control (MOPC-21) #555749 were from BD Biosciences (Franklin Lakes, NJ, USA). PKH26 # PKH26GL-1KT, Crystal Violet Solution 1%, aqueous solution # V5265, and Lipopolysaccharide (LPS) #437620 were from Sigma-Aldrich (St. Louis, Missouri (MO). CellTiter-Glo® Luminescent Cell Viability Assay # G7572 and ProFection® Mammalian Transfection System #E1200 were from PROMEGA (Madison, WI, USA). Cisplatin # 2251 and Gemcitabine hydrochloride #3259 were from Tocris Bioscience (Bristol, UK). TGF-β1, TGF-β2, TGF-β3; #171W4001M, Bio-Rad Laboratories (Hercules, CA, USA). Bafilomycin A1 #BML-CM110 (Enzo Life Sciences, Farmingdale, NY, USA) and TGF-β antibody (1D11) #GTX60614 (GeneTex, Irvine, CA, USA). Human IL-4 # GFH9-100, human IL-13 #GFH85-100, and Human TGF-β1 #GFH39-100 were from Cell Guidance System (Cambridge, UK). Alexa Fluor™ 647 Mouse IgG1 κ Isotype Control #557732 and Alexa Fluor^™^ 647 Mouse anti-Ki67 were from BD Pharmingen ^TM^ #571579 (San Diego, CA, USA).

### NSCLC spheroids and THP1 cells

NSCLC spheroids were generated as previously described [19, 20] by culturing surgically resected tumor tissues in serum-free medium supplemented with 20 ng/mL EGF and 10 ng/mL b-FGF (PeproTech, London, UK). Multicellular spheroids were passaged weekly and used for in vitro and in vivo studies within the first 12 passages. To limit cell adhesion and favor the formation of spheroids, cultures were maintained in non-adherent culture flasks (Greiner bio-one, Kremsmünster, Austria). Early-passage stocks were periodically thawed to prevent the accumulation of culture-related changes. Spheroids were routinely evaluated for their tumorigenic potential by assessing their ability to generate tumors that were histologically identical to the patient’s original lesion when injected into NOD. Cg-Prkdcscid Il2rgtm1Wjl/SzJ (NSG) mice (The Jackson Laboratory, Bar Harbor, ME, USA) [19]. THP-1 cells were obtained from the American Type Culture Collection (ATCC Manassas, VA USA) and maintained in DMEM (Thermo Fisher Scientific, Waltham, MA, USA) supplemented with 10% heat-inactivated fetal bovine serum (Cytiva, Marlborough, MA, USA), 100 U/mL penicillin, and 10 μg/mL streptomycin (Thermo Fisher Scientific) at 37 °C in a humidified 5% CO₂ atmosphere. All cultured cells, including both primary and commercially sourced lines, were routinely screened for mycoplasma contamination using the PCR Mycoplasma Test Kit (PanReac AppliChem ITW Reagents S.R.L. Italy) and checked by short tandem repeat (STR) (Applied Biosystems, Thermo Fisher Scientific, Waltham, MA, USA) for individual identity (data not shown).

### Reverse-phase protein microArrays (RPPA)

For RPPA experiments, NSCLC spheroids and the corresponding parental tumors obtained by laser-capture microdissection from frozen tumor sections were lysed in buffer containing T-PER reagent (Thermo Fisher Scientific), 300 mM NaCl, protease and phosphatase inhibitor cocktails (Sigma-Aldrich), and stored at –80 °C. Protein lysates were thawed on ice, quantified using the Bradford assay (Bio-Rad), and diluted to a final concentration of 0.5 mg/mL using 2X Tris-Glycine SDS sample buffer (Thermo Fisher Scientific), T-PER reagent, and 5% TCEP (Thermo Fisher Scientific). Samples were boiled for 3 min and stored at –80 °C until further use. Prior to printing, lysates were thawed to room temperature, boiled again for 3 min, and printed into nitrocellulose-coated slides using a robotic arrayer (Quanterix). Each sample was printed in technical triplicate as undiluted and 1:4 dilution pairs. Reference standard lysates (HeLa+Pervanadate, A431 + EGF, Jurkat+Etoposide, and Jurkat+Calyculin A) were included as positive and quality controls and printed in 10-point serial dilutions in triplicate at 0.5 mg/mL. A subset of slides was stained with Sypro Ruby (Thermo Fisher Scientific) to assess total protein content, while the remaining slides were stored desiccated at -20°C. Before immunostaining, slides were treated with Reblot Mild Solution (Merck Millipore), washed, and blocked with I-Block (Thermo Fisher Scientific) and Tween-20 (Sigma-Aldrich). Immunodetection was performed using a tyramide–biotin signal amplification system with appropriate secondary antibodies and IRDye®-680LT (LICORbio) fluorophore. Negative control slides incubated with secondary antibody only were included. Slides were scanned at 5 μm resolution using a Tecan Power Scanner™ (Tecan), and images were analyzed with MicroVigene software for spot detection, background subtraction, replicate averaging, and total protein normalization. RPPA data analysis was performed in R, using standard and custom scripts for quality control, internal normalization, and hierarchical clustering (packages: base, tidyverse, ComplexHeatmap, dendextend, data.table). A complete list of antibodies used is provided in Table S2.

### Lentiviral infection

AC and SCC spheroid cells were stably transduced with the previously described Tween-GFP lentiviral vector [58] using ProFection® Mammalian Transfection System (Promega Madison, WI, USA) following manufacturer’s instructions.

### Transwell and NIDO cultures

To investigate the interaction between macrophages and tumor spheroids, cells were pre-treated as follows. The THP-1 monocyte cell line (ATCC) was differentiated into macrophages using 10 ng/mL Phorbol 12-Myristate 13-Acetate (PMA) (Sigma-Aldrich, St Louis, MO, USA) dissolved in RPMI (Thermo Fisher Scientific) supplemented with 10% FBS for 24 h. During this step, cells were already distributed into the respective experimental conditions. For the transwell setup, approximately 4 × 10^5^ THP-1 cells were seeded into the cell culture insert equipped with a 0.4 μm pore-size membrane in a 6-well format (Corning^®^ Sigma Aldrich), whereas for the NIDO condition, 12 × 10^6^ THP-1 cells were plated in Petri dishes 100 × 20 mm (Corning® Falcon® Sigma Aldrich). The following day, the complete PMA medium was removed, and the cells were washed twice with PBS (Thermo Fisher Scientific). All cells, including the controls, were then reconditioned in the tumor stem cell medium used for the spheroids. We tested different macrophage: tumor cell ratios (0.5:1, 0.25:1, 1:1, 1:0.5 and 1:0.25) to identify a realistic condition for NIDO culture setup. A 1:1 ratio was established and subsequently used for all subsequent experiments. The same MAC: NSCLC ratio selected for NIDO cultures was also used for transwell experiments. Spheroids previously transduced with the Tween Luc-GFP vector were added at a 1:1 ratio either to the bottom compartment of the wells containing the transwell inserts or directly to the Petri dishes containing the differentiated macrophages. After 24 h, the medium from each condition was collected for ELISA analysis. Macrophages in the control and transwell setups were detached using TrypLE™ Express Enzyme (Thermo Fisher Scientific), whereas spheroid cells were collected directly. For NIDO cultures, the non-adherent fraction was collected first, followed by detachment of the remaining cells with TrypLE™ Express. The entire NIDO population was subsequently sorted. All samples from the different experimental settings were then prepared for subsequent analysis.

### Flow cytometry and cell cycle analysis

To evaluate the expression of stemness-related factors, dissociated AC and SCC spheroids were labeled with PE-conjugated primary antibodies against CD166 (1:25) and CD55 (1:25) (BD Biosciences Franklin Lakes, NJ, USA) for 30 min on ice, and subsequently cells were analyzed by CytoFLEX LX flow cytometer (Beckman Coulter, Brea, CA, USA) equipped with a CytExpert software (Beckman Coulter Life Science, Milan, Italy). The cell cycle status of control, transwell, and NIDO spheroids was assessed by staining dissociated spheroids with 50 µg/mL propidium iodide in a buffer containing 0.1% trisodium citrate, 9.65 mM NaCl, 0.1% NP-40, and 200 µg/mL RNase (Sigma-Aldrich) for 1 h at room temperature. The analysis of the cell cycle (the quantification of the percentage of cells in each cell cycle phase) was performed using Kaluza software (Beckman Coulter). Previously infected SCC and AC spheroids, along with macrophages, were separated by cell sorting. Macrophages were stained with CD45-PE (Miltenyi Biotec, Bergisch Gladbach, Germany), and GFP-positive AC and SCC spheroids were sorted with a MoFlo Astrios EQ 4-laser cell sorter (Beckman Coulter, Brea, CA, USA). SYTOX^TM^ Blue nucleic acid stain was used for dead cell exclusion. For Ki67 staining of AC cells co-cultured with macrophages at different concentrations, the procedure was performed as follows. Cells obtained from the co-culture, containing GFP-positive AC cells and GFP-negative macrophages, were first incubated with the viability dye SYTOX^TM^ Blue for 5 min on ice. The cells were then washed with PBS and fixed with 4% paraformaldehyde (PFA, Thermo Scientific) for 15 min. After fixation, they were washed with PBS containing 5% BSA and permeabilized with PBS containing 0.1% Triton X-100. Following an additional washing step, cells were incubated for 1 h on ice with the Alexa Fluor™ 647 Mouse IgG1, PE Mouse IgG2a κ Isotype Control, or primary antibodies Alexa Fluor™ 647 Mouse anti-Ki67 and CD45-PE (Miltenyi Biotec, Bergisch Gladbach, Germany), the latter used to stain macrophages. The analysis was performed only on GFP positive/CD45-negative cells by a CytoFLEX LX flow cytometer (Beckman Coulter, Brea, CA, USA) equipped with CytExpert software (Beckman Coulter Life Science, Milan, Italy).

### PKH26 staining

Macrophages were labeled with PKH26 (Sigma-Aldrich) for 2 min at 37 °C following the manufacturer’s instructions, and then extensively washed with PBS. PKH26 labeling efficiency was evaluated by flow cytometry, and cells were used for subsequent experiments only when ≥98% of the population was positive.

### Time-lapse microscopy

To qualitatively and quantitatively analyze the interactions between macrophages and tumor spheroids in NIDO cultures, PKH26-positive macrophages (4 × 10⁵) were allowed to adhere in 6-well plates (Corning) and subsequently exposed to AC spheroids (4 × 10⁵) or cultured alone. Time-lapse imaging was performed using a Zeiss LSM 900 confocal laser scanning microscope (Carl Zeiss, Germany) equipped with an Ibidi incubation system (Ibidi, Germany) for CO₂ and temperature regulation. Images were acquired every 3 min (for a total of 8.055 min) with an Axiocam 702 camera (Carl Zeiss, Germany) using a 20× objective. Acquired images were analyzed by the Zen Blue (3.2) software (Carl Zeiss, Germany), and morphological parameters and the time of interaction of NIDO were evaluated by ImageJ software at representative time points extracted from the time-lapse recording. A 125-h time-lapse video was analyzed to quantify interactions between stem cells and macrophages. Interactions were classified as: (i) transient (brief contacts within blocks of 50 frames), (ii) persistent (lasting ≥500 frames), and (iii) total (sum of transient and persistent). Pearson’s correlation test was used to evaluate the temporal trend of total interactions.

### Clonogenicity assay

For clonogenic assays, dissociated spheroids were plated in duplicate in non-adherent 24-well plates (Corning) at a density of 500 cells per well. Cells were suspended in 0.3% agarose layered over 0.4% SeaPlaque^®^ Agarose (Lonza Bioscience, Walkersville, MD, USA) and incubated at 37 °C in a humidified atmosphere with 5% CO₂. Colony formation was assessed 28 days after seeding. Colonies were stained with crystal violet (0.1% in 10% methanol) (Sigma-Aldrich) and categorized as follows: small (30–60 μm), medium (60–90 μm), and large ( > 90 μm). One day after plating, an initial cell count was performed. Clonogenic efficiency was calculated as the ratio between colonies and the number of cells initially seeded, while colony size distribution was expressed relative to the total number of colonies. Data are reported as the percentage of colonies formed relative to the number of cells plated.

### Immunofluorescence analysis of cultured cells

For immunofluorescence experiments, cell cultures were processed as follows. For all three experimental setups (control, transwell, and NIDO), monocytes were differentiated into macrophages directly in 24-well plates (Corning) in the presence of the polylysine-coated slides (13 mm, Thermo Fisher Scientific). In the NIDO condition, after 48 h most of the culture medium was removed, and the remaining cell suspension, containing adherent macrophages and partially floating spheroids, was centrifuged within the plate to promote the attachment of the suspended cells to the coated slide. At the end of the procedure, all slides were fixed with 4% PFA (Thermo Fisher Scientific), permeabilized with 0.1% Triton X-100 (Bio-Rad, Hercules, CA, USA), and then incubated overnight at 4 °C with the following primary antibodies: TTF1, CD63, cytokeratin 7, cytokeratin 5/6, CD68 (Santa Cruz), Ki67 and TGF-β1 (Novus Biologicals) (see Antibodies and Reagents section). After two washes in PBS, cells were incubated with the appropriate secondary antibodies such as donkey anti-rabbit Alexa Fluor™ 488 and donkey anti-mouse Alexa Fluor™ 555 (Thermo Fisher Scientific) for 30 min at room temperature in the dark. Slides were then stained for 15 min with 4,6-diamidino-2-phenylindole (DAPI) and phalloidin Alexa Fluor™ 647 (Thermo Fisher Scientific) for nuclear and cytoskeletal staining, diluted in PBS containing 3% BSA, and mounted with ProLong™ Gold Antifade (Thermo Fisher Scientific). Slides were analyzed using a Zeiss LSM 900 confocal laser scanning microscope (Carl Zeiss, Germany), and acquired images were processed with ZEN Blue software (version 3.2; Carl Zeiss, Germany).

### Immunofluorescence analysis of FFPE tumor sections

Formalin-fixed and paraffin-embedded (FFPE) AC and SCC tumor sections were analyzed as follows. Sections were cut (thickness: 5 µm) from FFPE surgical NSCLC tumor samples, deparaffinized, hydrated, and then incubated in citrate buffer (SIGMA) for antigen retrieval in a 96 °C water bath for 10 min. Tissues were washed in PBS and incubated with glycine 1 M for 1 h. Sections were incubated with primary antibodies anti-CD68 (1:100) and anti-Pan CK (1: 100) (Dako) in PBS containing 3% BSA, 3% FBS, and 0,1% Triton X-100 overnight at 4 °C. After washing in PBS, sections were incubated with donkey anti-mouse Alexa Fluor™ 647 and donkey anti-rabbit Alexa Fluor™ 488-conjugated secondary antibodies (Thermo Fisher Scientific) for 30 min at room temperature in the dark. Slides were then stained for 15 min with 4,6-diamidino-2-phenylindole (DAPI) diluted in PBS containing 3% BSA (Thermo Fisher Scientific) for nuclear staining and mounted with ProLong™ Gold Antifade (Thermo Fisher Scientific). Slides were analyzed using a Zeiss LSM 900 confocal laser scanning microscope (Carl Zeiss, Germany), and images were processed with ZEN Blue software (version 3.2; Carl Zeiss, Germany).

### ELISA

Quantification of the three TGF-β isoforms (TGF-β1, TGF-β2, TGF-β3; #171W4001M, Bio-Rad Laboratories, Hercules, CA, USA) in conditioned media was achieved by xMAP technology through a Luminex platform (Bio-Rad) equipped with a magnetic washer workstation according to the manufacturer’s protocol. The quantification was carried out on 50 µl of conditioned medium with a Bio-Plex array reader (Bio-Plex 200 System) and Bio-Plex Software Manager (Version 6.1, Bio-Rad). Briefly, the assay uses fluorescent magnetic beads coated with protein-specific antibodies that bind the target proteins in the sample. After washing, biotinylated detection antibodies and a streptavidin–phycoerythrin conjugate form the final fluorescent complex. The reader measures bead fluorescence, and the mean fluorescence intensity (MFI) is converted to protein concentration. The analyte amount is proportional to the phycoerythrin signal. Results were expressed as pg/mL and were normalized to the total protein concentration of the sample [59].

### Cell viability assay

To evaluate the capacity of macrophages to confer chemoresistance to AC and SCC spheroids, single cells of control, transwell and NIDO cultures (3 ×10^3^ well) were seeded alone or in presence of chemotherapeutic agents (5–10 μg/mL cisplatin, 25μM gemcitabine) in 96-well plates (three replicates per experimental point) in the appropriate medium and incubated in a humidified atmosphere at 37 °C, 5% CO_2_ for 48 h. To assess whether TGF-β stimulation on NSCLC spheroids mimics the effects induced by macrophages, cells were treated with TGF-β at a concentration of 100 nM for 24 or 48 h, either alone or in combination with the chemotherapeutic agents described above. To assess the effectiveness of Bafilomycin A1 (BafA) (Enzo Life Sciences) and TGF-β neutralizing antibody (1D11, GeneTex) in restoring sensitivity to chemotherapeutic agents after NIDO culture, cells were treated as follows. Macrophages were incubated for 2 h with BafA (6 nM) or MoAb 1D11 (4,62 μg/mL). Tumor spheroids were then added to the Petri dishes containing macrophages, together with their respective complete culture media: control, BafA-supplemented, or 1D11-supplemented medium. After 48 h, the cells were separated by sorting and plated as previously described in the presence of the chemotherapeutic agents. The viability of NSCLC spheroids was determined by CellTiter-GLO^TM^ luminescent cell viability assay (Promega) according to the manufacturer’s directions. Luminescence was detected with an Envision 2105 multimode plate reader with an alpha 680 nm laser (Perkin Elmer, Waltham, MA, USA).

### Western blotting

Spheroids and macrophages were lysed in lysis buffer (1% NP40 lysis buffer: 20 mM Tris HCl pH 7.2, 200 mM NaCl, 1% NP40, supplemented with protease inhibitor/phosphatase inhibitor cocktails I and II from Sigma-Aldrich). The protein concentration in lysates was determined by Bradford assay (Bio-Rad Laboratories). Equal amounts of proteins were run on a 4–12% precast gel (Thermo Fisher Scientific) and then transferred to nitrocellulose membranes (GE Healthcare Life Sciences). Blots were blocked with TBST 5% non-fat dry milk (Bio-Rad Laboratories) and incubated overnight at 4 °C with primary antibodies diluted in TBST/BSA 5%. After three washes in TBST, blots were incubated for 45 min with specific secondary HRP-conjugated antibodies (Thermo Fisher Scientific) dissolved in TBST/BSA 5%. Chemiluminescent signals were detected with Amersham ECL Prime or Select western blotting detection reagent (GE Healthcare Life Sciences). Immunoblotting images were recorded and analyzed by Bio-Rad ChemiDoc Imagers (Bio-Rad Laboratories, Hercules). Immunoblot densitometry quantification was performed by ChemiDocMP (Bio-Rad Laboratories), and signal intensity was quantified with the Image Lab software. Normalization was performed using antibodies against β-actin, β-tubulin, or GAPDH (from Merck) as reference standards. Primary and secondary antibodies used for western blotting are indicated in the antibodies and reagents section. Full-length western blots are provided in the Supplementary Information section as Supplementary file 1 original western blot.

### Real-time PCR

Total RNA was extracted with TRIzol (Thermo Fisher Scientific) following the manufacturer’s instructions. 1 μg of RNA was reverse transcribed with M-MLV reverse transcriptase (Thermo Fisher Scientific), and 50 ng of cDNA was used as a template in the PCR reactions. Specific primers were used for CRIPTO (Forward: 5’-ACAGAACCTGCTGCCTGAAT-3’, Reverse: 5’-ATCACAGCCGGGTAGAAATG-3’) while a TaqMan assay (Integrated DNA Technologies, IDT, Coralville, USA) was used for NANOG (Hs.PT.58.21480849), POU5F1 (Hs.PT.58.14494169.g), SOX2 (Hs.PT.58.237897.g), TGFB1 (Hs.PT.58.39813975), IL12B (Hs.PT.58.2925830), NOS2 (Hs.PT.58.14740388), TNF (Hs.PT.58.45380900), CCR7 (Hs.PT.58.1402597), MRC1(Hs.PT.58.15093573), IL10 (Hs.PT.58.2807216), VIMENTIN (Hs.PT.58.38906895), E-CADHERIN (Hs.PT.58.3324071), SNAI1 (Hs.PT.58.2984401), SNAI2 (Hs.PT.58.1772559), ZEB1 (Hs.PT.58.39178574) and ZEB2 (Hs.PT.58.1089006). Normalization was performed using GAPDH (Forward: 5’-TGGAAGATGGTGATGGGATT-3’, Reverse: 5’-GAGTCAACGGATTTGGTCGT-3’) as a reference for CRIPTO and using β-actin (Hs.PT.39a.22214847) as a reference for the TaqMan assay. Values are expressed in terms of 2^–^^ΔΔCT^ where ΔΔCT=ΔCTsample−ΔCTcalibrator. ΔCT is the difference in threshold cycles between the specific RNA and reference amplicons, and CT is a parameter given by StepOne Plus Real-Time PCR software by negative correlation with an internal reference dye (ROX).

### Statistical analyses

Data are shown as mean ± SD. Statistical significance was determined by one-tailed Student’s *t* test as indicated in the corresponding figure legends. The number of biological repeats is indicated in the corresponding figure legends. Statistical analysis was performed using R (version 4.5.3; R Core Team, 2026). Statistical significance is indicated as *P < 0.05, **P < 0.01, ***P < 0.001, and n.s. = not significant. Pearson correlation test was performed to evaluate the temporal trend of total interactions.

Assuming an average pooled SD of 10% of the group mean, Cohen’s d was calculated as the expected between-group difference divided by the pooled SD. Sensitivity analyses using a one-sided two-sample t-test, α = 0.05, 80% power, and equal group sizes indicated that *n* = 3 biological replicates per group allow detection of Cohen’s d ≥ 2.48, corresponding to a minimum detectable difference of approximately 25%, whereas *n* = 5 biological replicates per group allow detection of Cohen’s d ≥ 1.72, corresponding to a minimum detectable difference of approximately 17%. Since preliminary data indicated expected between-group differences of approximately 30% or greater, corresponding to d ≥ 3.0 with a pooled SD of 10%, *n* = 3–5 biological replicates per condition were considered adequate for detecting the expected large directional effects.

### Ethics statement

The study was conducted according to the guidelines of the Declaration of Helsinki and approved by the Institutional Review Board (Prot. AOO-ISS-31/03/2023-0015937). Written informed consent was obtained from all participants involved in the study. Human samples were collected and used in accordance with the approved study protocol and institutional ethical standards.

## Supplementary information

Supplementary table S1

Supplementary table S2

Supplementary Video S1

Supplementary Information

Supplementary file 1: original western blot

## Data Availability

The data supporting the findings of this study are available within the article and its Supplementary Information files. Additional data are available from the corresponding author upon reasonable request.

## Glossary

NSCLC: non-small-cell lung cancer
CSCs: cancer stem cells
EMT: epithelial-to-mesenchymal transition
TGF-β: Transforming Growth Factor-β
NIDO: Niche-Induced DOrmancy
AC: adenocarcinoma
SCC: squamous cell carcinoma
LCC: large cell carcinoma
TME: tumor microenvironment
TAMs: Tumor-associated macrophages
M1: pro-inflammatory/antitumorigenic
M2: anti-inflammatory/protumorigenic
THP-1: Tohoku Hospital Pediatrics-1
EGF: Epidermal Growth Factor
b-FGF: Basic Fibroblast Growth Factor
STR: Short Tandem Repeat
RPPA: Reverse-Phase Protein Microarrays
GFP: green fluorescent protein
ELISA: enzyme-linked immunosorbent assay
CD166: Activated Leukocyte Cell Adhesion Molecule, ALCAM
CD55: Complement Decay-Accelerating Factor
CD45: Leukocyte Common Antigen, LCA
PKH26: Red Fluorescent Cell Linker
FFPE: Formalin Fixed and Paraffin Embedded
DIC: Differential Interference Contrast
