# Supplementary material for: Tumor plasticity in macrophage niches promotes dormancy, EMT and chemoresistance of non-small cell lung cancer stem cells
Source: Cell Death Discov. 2026 Jul 31;12:428. doi: 10.1038/s41420-026-03261-1 (PMC13714290; doi:10.1038/s41420-026-03261-1)
Supplement: Supplementary file 1 [file 41420_2026_3261_MOESM1_ESM.pdf]

Patient ID	Tumor Type	KRAS	EGFR	EML4-ALK Fusion	PTEN	PI3KCA	HER2
AC1	AC (pT4pN1IIIA-G3)	Mut G12C (ggt>tgt)	WT	No	WT	WT	WT
AC2	AC (pT2pN2pMx-IIIA-G2)	WT	WT	No	WT	WT	WT
SCC1	SCC (pT3pN0pMx-IIB-G3)	WT	WT	No	WT	WT	WT
SCC2	SCC (pT2N0-IB)	Mut G12C (ggt>tgt)	WT	No	WT	WT	WT

Table S1. Clinical staging and classification of lung cancer samples and mutational status of lung cancer-associated genes.
