# Supplementary material for: Tumor plasticity in macrophage niches promotes dormancy, EMT and chemoresistance of non-small cell lung cancer stem cells
Source: Cell Death Discov. 2026 Jul 31;12:428. doi: 10.1038/s41420-026-03261-1 (PMC13714290; doi:10.1038/s41420-026-03261-1)
Supplement: Supplementary file 2 [file 41420_2026_3261_MOESM2_ESM.pdf]

RPPA_Antibody name	Company	Cat #	Company name	Host	Dilution
Actin-beta	Cell Signaling Technology	4967	Actin, Beta	R	1:1000
HER2	DAKO	A0485	c-ErbB2/HER2	R	1:100
HSP90	Cell Signaling Technology	4875	HSP90 (E289)	R	1:1000
4E-BP1_pS65	Cell Signaling Technology	9451	Phospho-4E-BP1 (S65)	R	1:50
Adducin_pS662	Upstate	06-820	Phospho-Adducin (S662)	R	1:100
AKT_pS473	Cell Signaling Technology	9271	Phospho-Akt (S473)	R	1:100
AKT_pT308	Cell Signaling Technology	9275	Phospho-Akt (T308)	R	1:50
ASK1_pS83	Cell Signaling Technology	3761	Phospho-ASK1 (S83)	R	1:50
BAD_pS112	Cell Signaling Technology	9291	Phospho-Bad (S112)	R	1:200
BAD_pS136	Cell Signaling Technology	9295	Phospho-BAD (S136)	R	1:50
Catenin-beta_pT41-pS45	Cell Signaling Technology	9565	Phospho-Catenin(beta) (T41/S45)	R	1:100
BCL2_pS70	Cell Signaling Technology	2871	Phospho-Bcl-2 (S70)	R	1:100
bRAF_pS445	Cell Signaling Technology	2696	Phospho-B-Raf (S445)	R	1:50
cABL_pT735	Cell Signaling Technology	2864	Phospho-c-Abl (T735)	R	1:50
cABL_pY245	Cell Signaling Technology	2861	Phospho-c-Abl (Y245)	R	1:100
Smac-Diablo	Cell Signaling Technology	2954	Smac/Diablo	M	1:1000
CHK2_pS33-35	Cell Signaling Technology	2665	Phospho-Chk-2 (S33/35)	R	1:50
cKIT_pY703	Zymed	34-9300	Phospho-cKit (Y703)	R	1:200
cKIT_pY719	Cell Signaling Technology	3391	Phospho-c-Kit (Y719)	R	1:50
CREB_pS133	Cell Signaling Technology	9191	Phospho-CREB (S133)	R	1:100
EGFR_pY992	Cell Signaling Technology	2235	Phospho-EGFR (Y992)	R	1:50
EGFR_pY1045	Cell Signaling Technology	2237	Phospho-EGFR (Y1045)	R	1:50
EGFR_pY1068	Cell Signaling Technology	2234	Phospho-EGFR (Y1068)	R	1:50
EGFR_pY1148	BioSource	44-792	Phospho-EGFR (Y1148)	R	1:100
EGFR_pY1173	BioSource	44-794	Phospho-EGFR (Y1173)	R	1:100
eIF-4G_pS1108	Cell Signaling Technology	2441	Phospho-eIF4G (S1108)	R	1:1000
eNOS_pS1177	Cell Signaling Technology	2571	Phospho-eNOS (S1177)	R	1:50
eNOS-NOSIII_pS116	Upstate	07-357	Phospho-eNOS/NOS III (S116)	R	1:500
ERK1-2_pT202-pY204	Cell Signaling Technology	9101	Phospho-ERK 1/2 (T202/Y204)	R	1:500
FADD_pS194	Cell Signaling Technology	2781	Phospho-FADD (S194)	R	1:50
FAK_pY576-77	Cell Signaling Technology	3281	Phospho-FAK (Y576/577)	R	1:100
FKHR_pS256	Cell Signaling Technology	9461	Phospho-FKHR (S256)	R	1:100
HER2_pY1248	Upstate	06-229	Phospho-ErbB2/HER2 (Y1248)	R	1:1000
FKHR-FKHRL1_pT24-pT32	Cell Signaling Technology	9464	Phospho-FKHR (T24)/FKHRL1 (T32)	R	1:200
GSK3alpha-beta_pY279-pY216	BioSource	44-604	Phospho-GSK-3alpha (Y279)/beta (Y216)	R	1:500
GSK3-beta_pS9	Cell Signaling Technology	9336	Phospho-GSK-3beta (S9)	R	1:1000
IRS-1_pS612	Cell Signaling Technology	2386	Phospho-IRS-1 (S612)	R	1:200
JAK1_pY1022	Cell Signaling Technology	3331	Phospho-Jak1 (Y1022/1023)	R	1:50
MARCKS_pS152-56	Cell Signaling Technology	2741	Phospho-MARCKS (S152/156)	R	1:50
MEK1-2_pS217-221	Cell Signaling Technology	9121	Phospho-MEK1/2 (S217/221)	R	1:500
MET_pY1234-35	Cell Signaling Technology	3126	Phospho-Met (Y1234/1235)	R	1:200
MSK1_pS360	Cell Signaling Technology	9594	Phospho-MSK1 (S360)	R	1:50
MTOR_pS2448	Cell Signaling Technology	2971	Phospho-mTOR (S2448)	R	1:100
MTOR_pS2481	Cell Signaling Technology	2974	Phospho-mTOR (S2481)	R	1:100
NF-kB_pS536	Cell Signaling Technology	3031	Phospho-NF-kappaB p65 (S536)	R	1:50
p27_pT187	Zymed	71-7700	Phospho-p27 (T187)	R	1:50
p38MAPK_pT180-pY182	Cell Signaling Technology	9211	Phospho-p38 MAP Kinase (T180/Y182)	R	1:50
p70S6K_pS371	Cell Signaling Technology	9208	Phospho-p70 S6 Kinase (S371)	R	1:50
p70S6K_pT389	Cell Signaling Technology	9205	Phospho-p70 S6 Kinase (T389)	R	1:100
p90RSK_pS380	Cell Signaling Technology	9341	Phospho-p90RSK (S380)	R	1:200
PAK1_pS199-204_PAK2_pS192-197	Cell Signaling Technology	2605	Phospho-PAK1 (S199/204)/PAK2 (S192/197)	R	1:50
PDGFR-beta_pY716	Upstate	07-021	Phospho-PDGF Receptor beta (Y716)	R	1:200
PDK1_pS241	Cell Signaling Technology	3061	Phospho-PDK1 (S241)	R	1:200
PKA-C_pT197	Cell Signaling Technology	4781	Phospho-PKA C (T197)	R	1:200
PKC-alpha_pS657	Upstate	06-822	Phospho-PKC alpha (S657)	R	1:1000
PKCalpha-betaII_pT638-41	Cell Signaling Technology	9375	Phospho-PKCa/B II (T638/641)	R	1:50
PKC-theta_pT538	Cell Signaling Technology	9377	Phospho-PKCtheta (T538)	R	1:100
PKCzeta-lambda_pT410-403	Cell Signaling Technology	9378	Phospho-PKC zeta/lambda (T410/403)	R	1:50
PLC-gamma1_pY783	Cell Signaling Technology	2821	Phospho-PLCgamma1 (Y783)	R	1:1000
PRAS40_pT246	Biosource	44-1100	Phospho-PRAS40 (T246)	R	1:1000
PTEN_pS380	Cell Signaling Technology	9551	Phospho-PTEN (S380)	R	1:500
BAD	Cell Signaling Technology	9292	Bad	R	1:1000
BAK	Cell Signaling Technology	3792	Bak	R	1:50
BAX	Cell Signaling Technology	2772	Bax	R	1:1000
FAK_pY397	Becton Dickinson	611806	Phospho-FAK (Y397)	M	1:50
Caspase3	Cell Signaling Technology	9662	Caspase-3	R	1:1000
Cleaved Caspase3_D175	Cell Signaling Technology	9661	Caspase-3, cleaved (D175)	R	1:50
Cleaved Caspase9_D315	Cell Signaling Technology	9505	Caspase-9, cleaved (D315)	R	1:50
Cleaved PARP_D214	Cell Signaling Technology	9541	PARP, cleaved (D214)	R	1:1000
STAT3_pY705	Upstate	05-485	Phospho-Stat3 (Y705)	M	1:50
HER3	Cell Signaling Technology	4754	ErbB3/HER3 (1B2)	R	1:1000
4E-BP1_pT70	Cell Signaling Technology	9455	Phospho-4E-BP1 (T70)	R	1:50
HER4	Cell Signaling Technology	4795	ErbB4/HER4 (111B2)	R	1:1000
Musashi	Cell Signaling Technology	2154	Musashi	R	1:500
PYK2_pY402	Cell Signaling Technology	3291	Phospho-Pyk2 (Y402)	R	1:200
RasGRF1_pS916	Cell Signaling Technology	3321	Phospho-Ras-GRF1 (S916)	R	1:50
S6RP_pS235-36	Cell Signaling Technology	4856	Phospho-S6 Ribosomal Protein (S235/236) (2F9)	R	1:200
SAP-JNK_pT183-pY185	Cell Signaling Technology	9251	Phospho-SAPK/JNK (T183/Y185)	R	1:50
SHC_pY317	Upstate	07-206	Phospho-Shc (Y317)	R	1:200
SMAD2_pS465	Cell Signaling Technology	3101	Phospho-SMAD2 (S465/467)	R	1:250
Histone-H3_pS10	Upstate	06-570	Phospho-Histone H3 (S10)	R	1:500
EGFR_L858R	Cell Signaling Technology	3197	EGFR L858R Mutant	RmAb	1:50
cRAF_pS338	Cell Signaling Technology	9427	Phospho-c-Raf (S338) (56A6)	RmAb	1:200
STAT1_pY701	Upstate	07-307	Phospho-Stat1 (Y701)	R	1:500
STAT3_pS727	Cell Signaling Technology	9134	Phospho-Stat3 (S727)	R	1:100
STAT5_pY694	Cell Signaling Technology	9351	Phospho-Stat5 (Y694)	R	1:50
PTEN	Cell Signaling Technology	9552	PTEN	R	1:1000
Tuberin-TSC2_pY1571	Cell Signaling Technology	3614	Tuberin/TSC2 (Y1571)	R	1:1000
IkB-alpha_pS32-36	Becton Dickinson	551818	Phospho-IkappaB-alpha (S32/36) (39A1431)	M	1:50
VEGFR2_pY996	Cell Signaling Technology	2474	Phospho-VEGFR 2 (Y996)	R	1:50
VEGFR2_pY1175	Cell Signaling Technology	2478	Phospho-VEGFR 2 (Y1175)	R	1:50
Survivin	Cell Signaling Technology	2808	Survivin (71G4)	RmAb	1:1000
Tubulin alpha-beta	Cell Signaling Technology	2148	Tubulin, a/B	R	1:1000
BMI1	Chemicon	MAB4376	Bmi-1 (10C7.2)	M	1:500
CD133	Miltenyi Biotec	130-092-3	CD133 (W6B3C1)	M	1:100
COX2	Becton Dickinson	610203	Cox-2 (33)	M	1:250
Estrogen Receptor-alpha	Cell Signaling Technology	2512	Estrogen Rec alpha (62A3)	M	1:1000
HSP70	Stressgen	SPA-810	HSP70 (C92F3A-5)	M	1:2000
Estrogen Receptor-alpha_pS118	Cell Signaling Technology	2511	Phospho-Estrogen Rec a (S118) (16JR)	M	1:1000

Table S2. RPPA antibodies
