# Supplementary material for: Tumor plasticity in macrophage niches promotes dormancy, EMT and chemoresistance of non-small cell lung cancer stem cells
Source: Cell Death Discov. 2026 Jul 31;12:428. doi: 10.1038/s41420-026-03261-1 (PMC13714290; doi:10.1038/s41420-026-03261-1)
Supplement: Supplementary file 3 [file 41420_2026_3261_MOESM4_ESM.pdf]

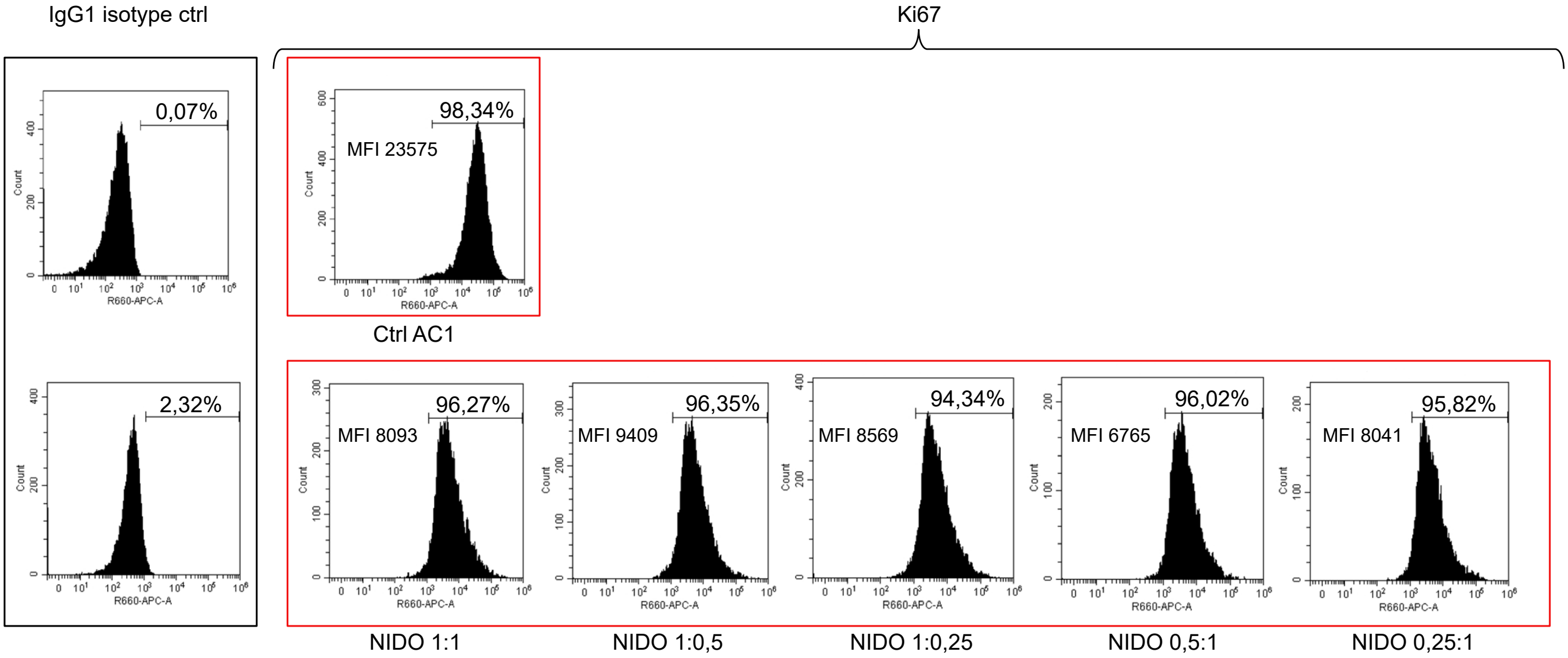

Fig. S1 Flow cytometry analysis of Ki67 levels in control and NIDO cultures produced with different ratios of macrophages:AC1 cells. Ratios are indicated below each plot. Each plot reports the percentage of Ki67-positive AC1 cells together with the corresponding mean fluorescence intensity (MFI). Ctrl AC1 cells were not co-cultured with macrophages. Black boxes indicate control and NIDO samples stained with Alexa Fluor™ 647-conjugated IgG1 isotype, while red boxes indicate the corresponding samples stained with Alexa Fluor™ 647-conjugated Ki67 antibody.

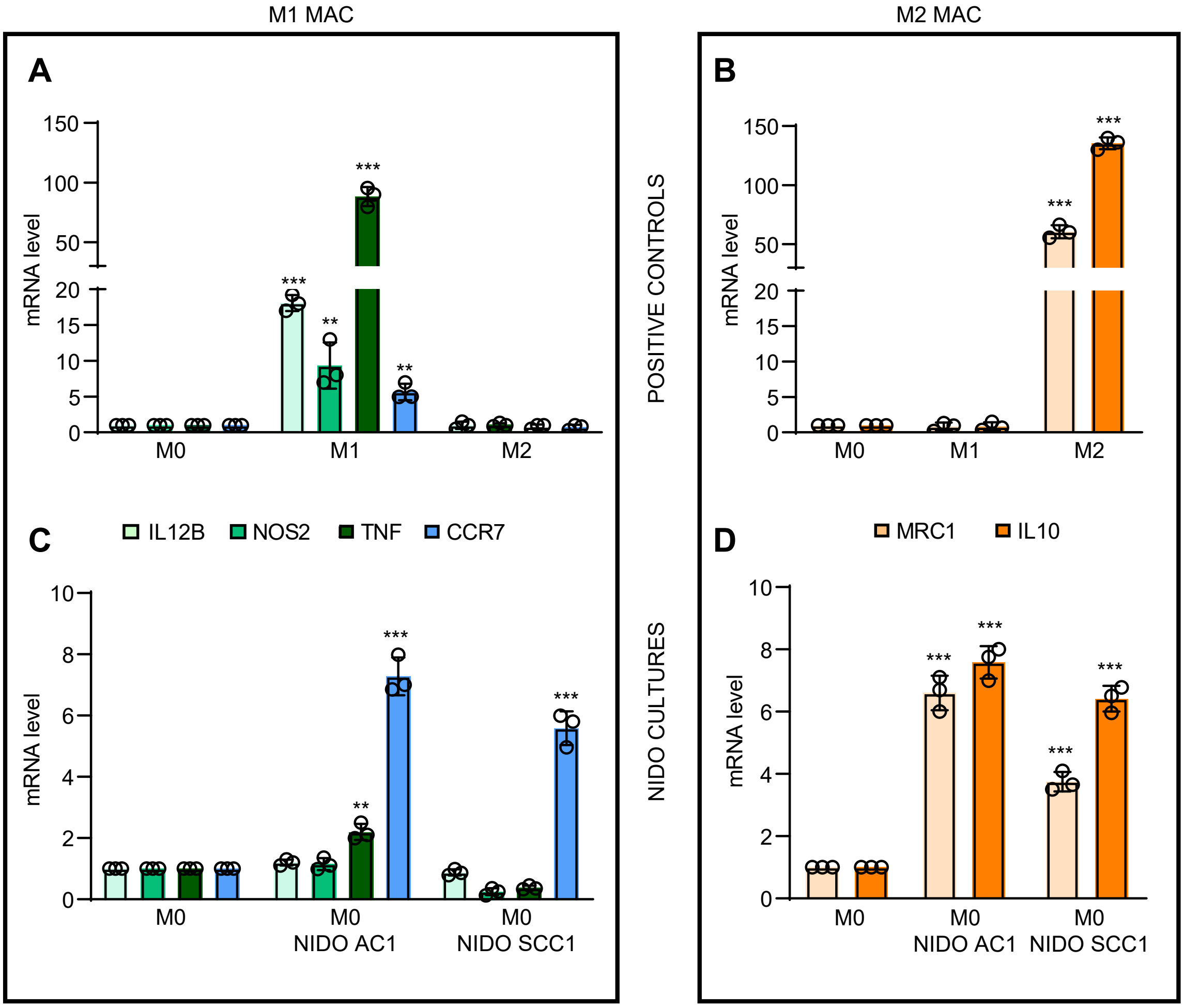

Figure S2. Macrophages polarization by NIDO cultures. Panels A and B (Positive controls) show the expression of M1 and M2 factors, respectively upon treatment with cytokines known to induce M1 or M2 polarization (LPS dose for M1 60 ng/mL, IL-4 and IL-13 dose for M2 25 ng/mL). Panels C and D show the expression of M1 and M2 factors in macrophages upon NIDO cultures with AC1 or SCC1. Data are presented as mean ±SD from n=3 independent experiments. Statistical significance: $**P < 0.01$ and $***P < 0.001$ (one-tailed t-test).

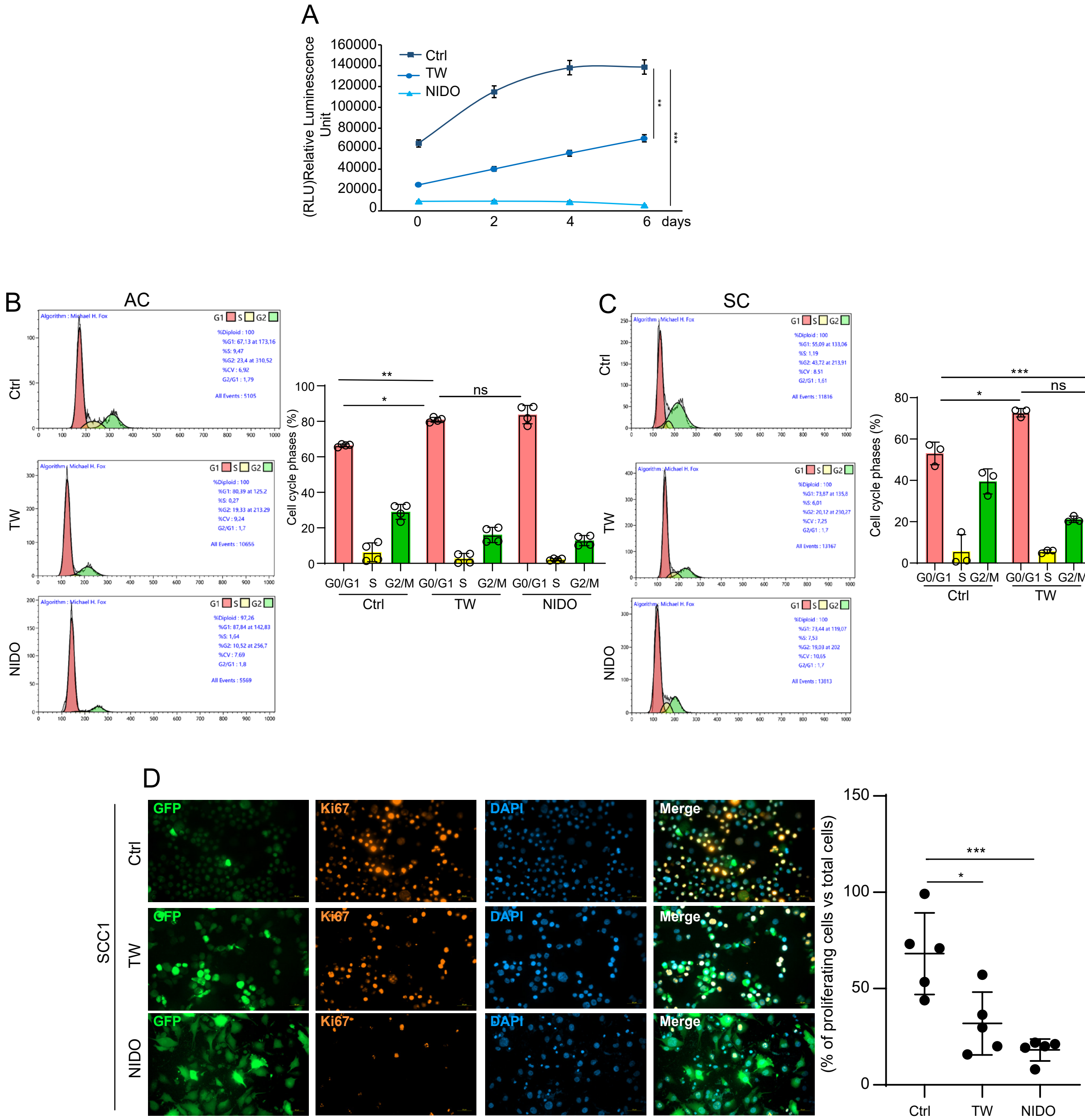

Figure S3. Effect of NIDO cultures on cell proliferation. **A** Results of CellTiter-Glo assay reported as RLU at different time points for control (dark blue square), transwell-derived SCC1 (TW, dark cerulean circle), and NIDO-derived SCC1 (light cerulean triangle). **B**-**C** Cell cycle analysis performed using FlowJo, identifying G0/G1 (pink), S (yellow), and G2/M (green) phases by Gaussian fitting of DNA content histograms in AC and SCC spheroids derived from control, transwell and NIDO conditions. Replicates are shown in the graphs on the right; color bars maintain the same colors as the corresponding cell cycle peaks. **D** Representing confocal images of GFP-positive SCC1 spheroids stained for the proliferation marker Ki67 (orange) and nuclei (DAPI, blue), derived from control, transwell and NIDO cultures (right). Quantitative analysis of Ki67-positive cells (area), normalized to GFP/DAPI-positive cells (area) and reported as percentages, was conducted using ImageJ software. Mean ±SD, data were obtained from three independent sets, each consisting of five fields per group. Statistical significance: *P < 0.05 and ***P < 0.001 (one-tailed t-test, n.s.= not significant).

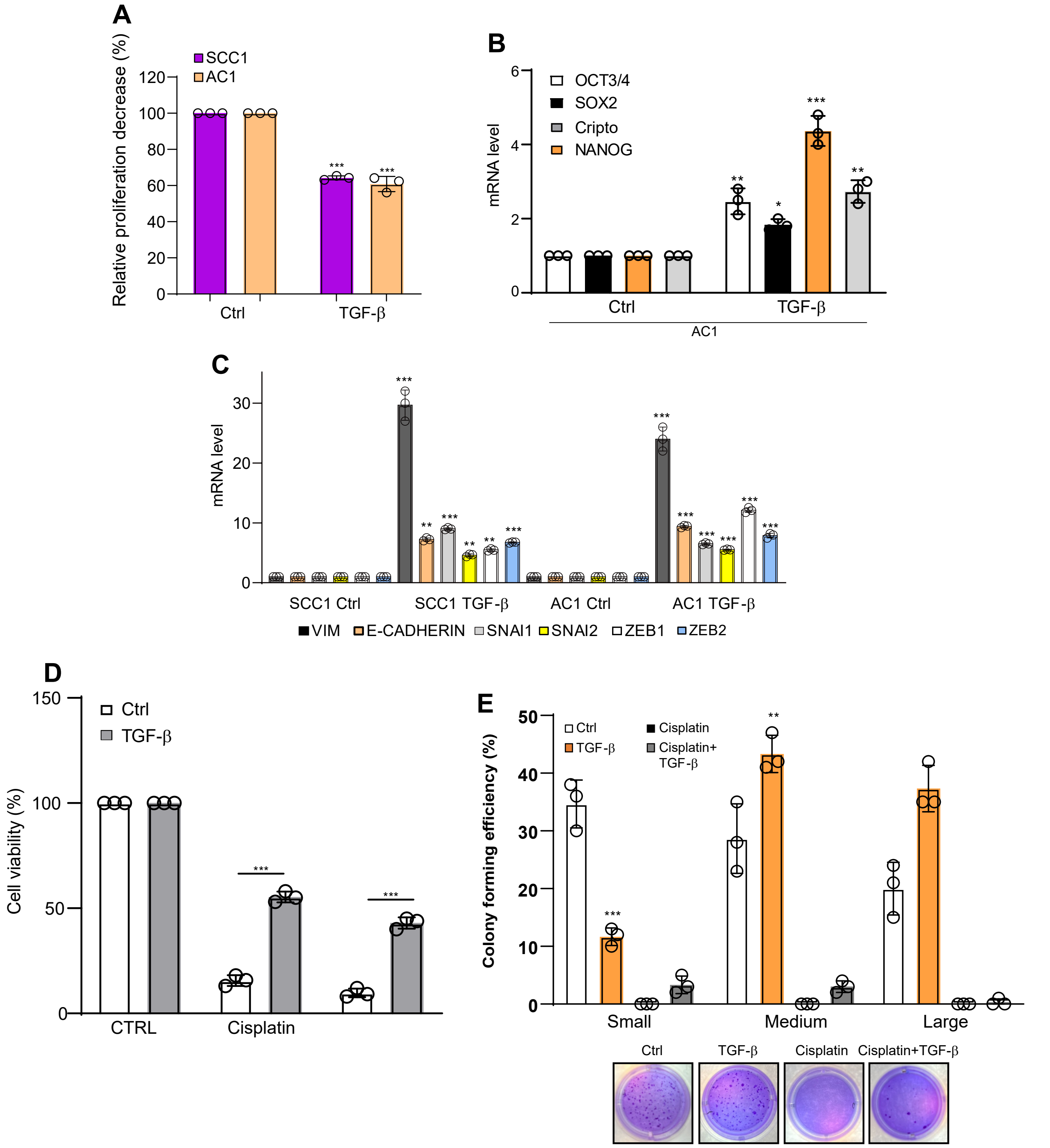

Figure S4. Effect of exogenous TGF-β on proliferation, stemness traits, and chemoresistance in spheroids. **A** proliferation assessed by CellTiter-Glo assay reported as RLU, after 24 hours of treatment with TGF-β (100 ng/mL) in SCC1 and AC1 spheroids. **B-C** qRT-PCR analysis of the indicated transcripts associated with stemness (OCT3/4, SOX2, NANOG and Cripto) and EMT-related factors (VIM, E-Cadherin, SNAI1, SNAI2, ZEB1 and ZEB2) performed in AC1 spheroids following 48 hours of TGF-β (100 ng/mL) treatment. **D** Cell viability assay performed on AC1 spheroids plated after 48 hours of TGF-β (100 ng/mL) pre-treatment to evaluate the induction of chemoresistance. Chemotherapeutic agents were used at the following concentrations: cisplatin (CIS, 10 μg/mL) and gemcitabine (GEM, 25 μM) for 48 hours. The graph shows the percentage of viable cells. Data are presented as mean ±SD of three technical replicates. **E** Upper panel: colony-forming assay performed on control and TGF-β (100 ng/mL) pre-treated spheroids, either alone or in combination with cisplatin (10 μg/mL), over 4 weeks. Results were evaluated as colony formation in semisolid culture after 4 weeks and expressed as normalized colony size or percentage relative to plated cells. Bottom panel representative soft agar images. Data are presented as mean ± SD of three technical replicates. Statistical significance: *P < 0.05, **P < 0.01 and ***P < 0.001 (one-tailed t-test).

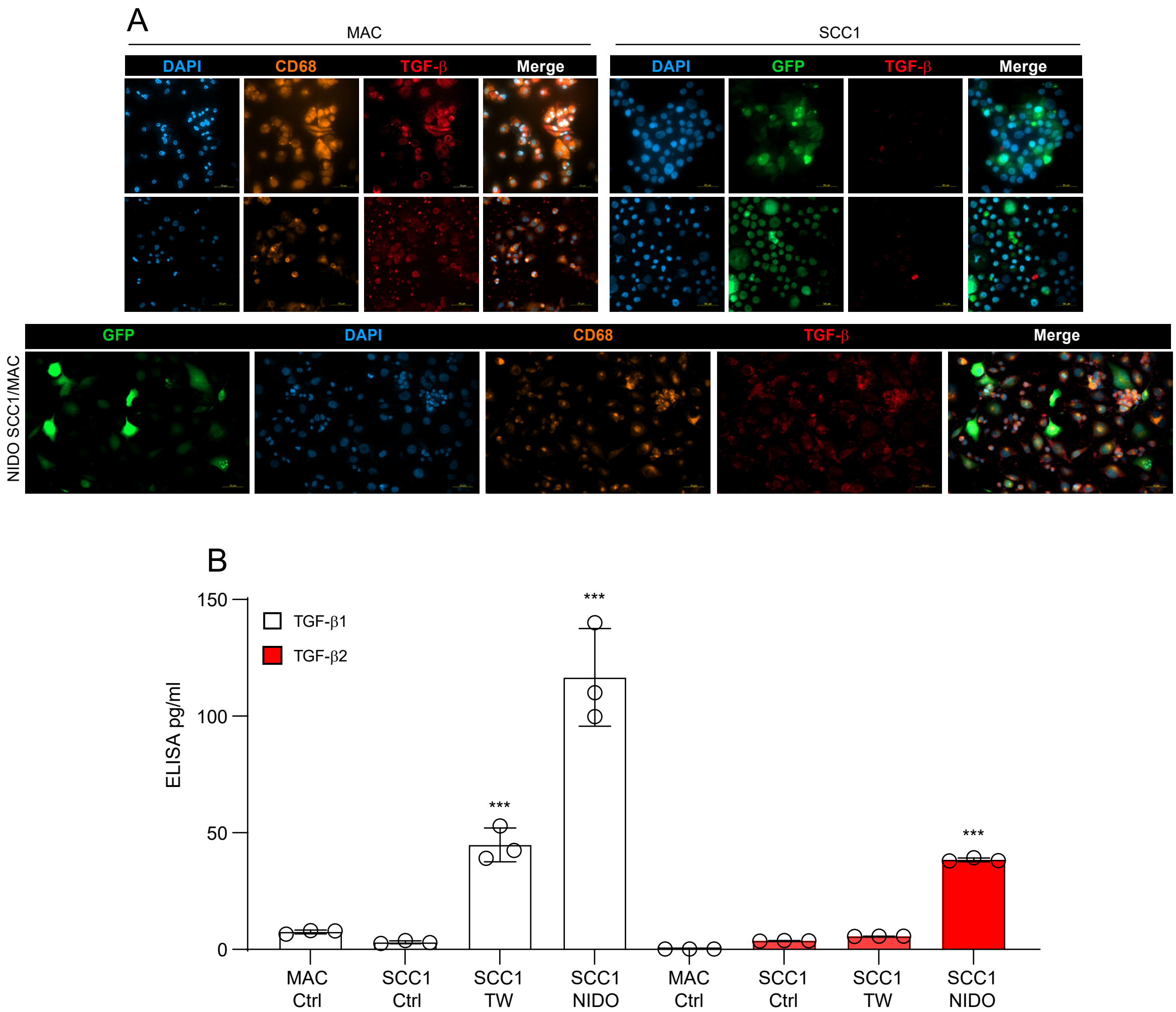

Figure S5. TGF-β expression in macrophages and SCC1 spheroids upon NIDO cultures. **A** representative confocal image of control, transwell and NIDO conditions. Upper left panel: macrophages were stained for CD68 (orange) and TGF-β (red). Upper right panel: GFP-positive SCC1 spheroids were stained for TGF-β (red). Bottom panel: NIDO images. Nuclei of both cell types were stained with DAPI (blue) (20x magnification, scale bar 20 μm). **B** The graph shows the analysis of secreted TGF-β1 and TGF-β2 levels detected by ELISA under different experimental conditions: macrophages and SCC1 spheroids control medium, transwell and NIDO cultures. Data are presented as mean ± SD of three technical replicates. Statistical significance: ***P < 0.001 (one-tailed t-test).

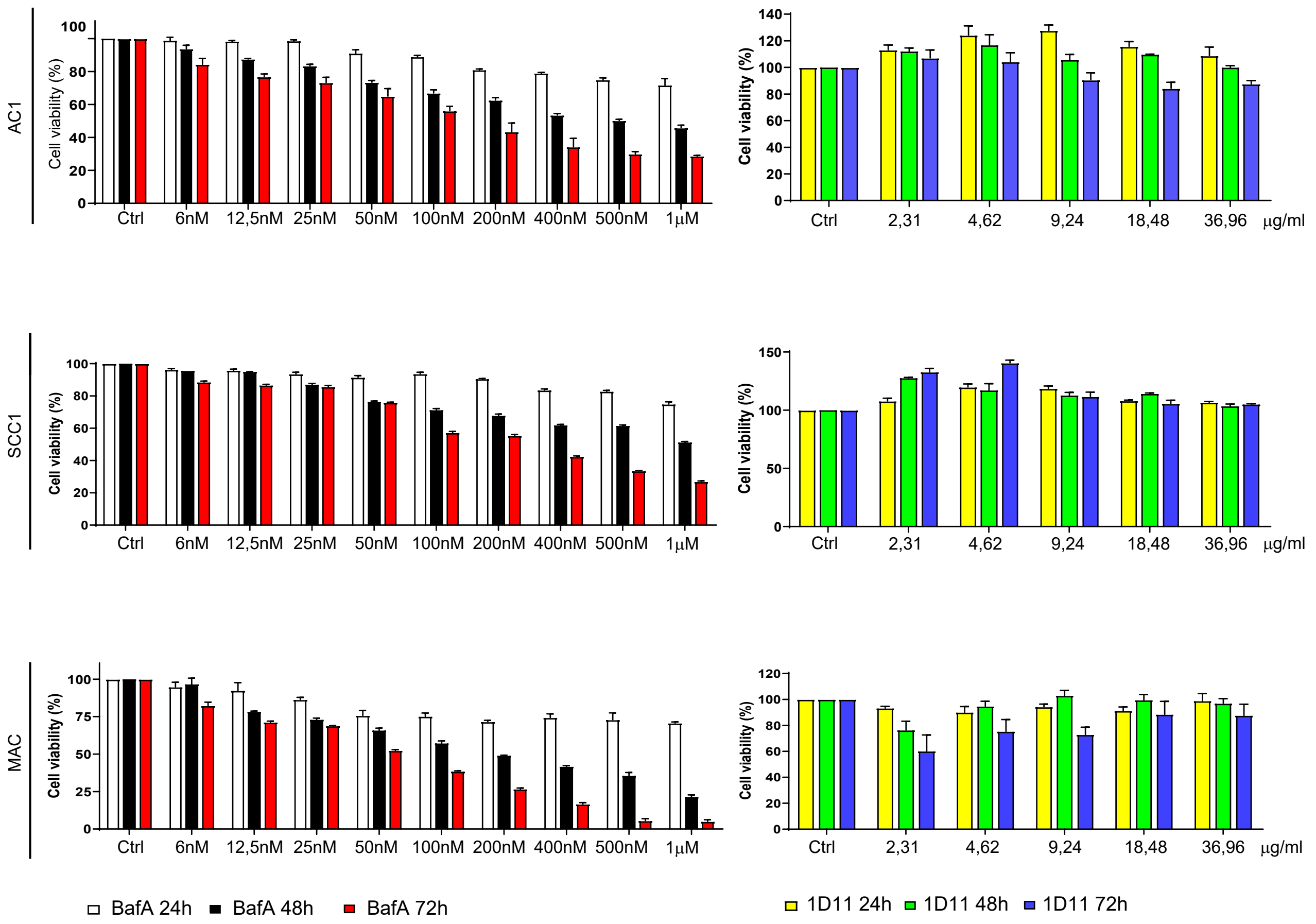

Figure S6. Identification of the lowest effective concentrationof BafA and TGF-β-neutralizing antibody (1D11) with no impact on cell viability. Cell viability assay was performed on AC1 and SCC1 spheroids and macrophages (MAC) treated with BafA (**A**, **C**, **E**) or 1D11 (**B**, **D**, **F**) at the indicated concentrations and time points. Data are presented as the mean ± SD of three technical replicates.
