# Supplementary material for: Tumor plasticity in macrophage niches promotes dormancy, EMT and chemoresistance of non-small cell lung cancer stem cells
Source: Cell Death Discov. 2026 Jul 31;12:428. doi: 10.1038/s41420-026-03261-1 (PMC13714290; doi:10.1038/s41420-026-03261-1)
Supplement: Supplementary file 4 [file 41420_2026_3261_MOESM5_ESM.pdf]

Figure 4D

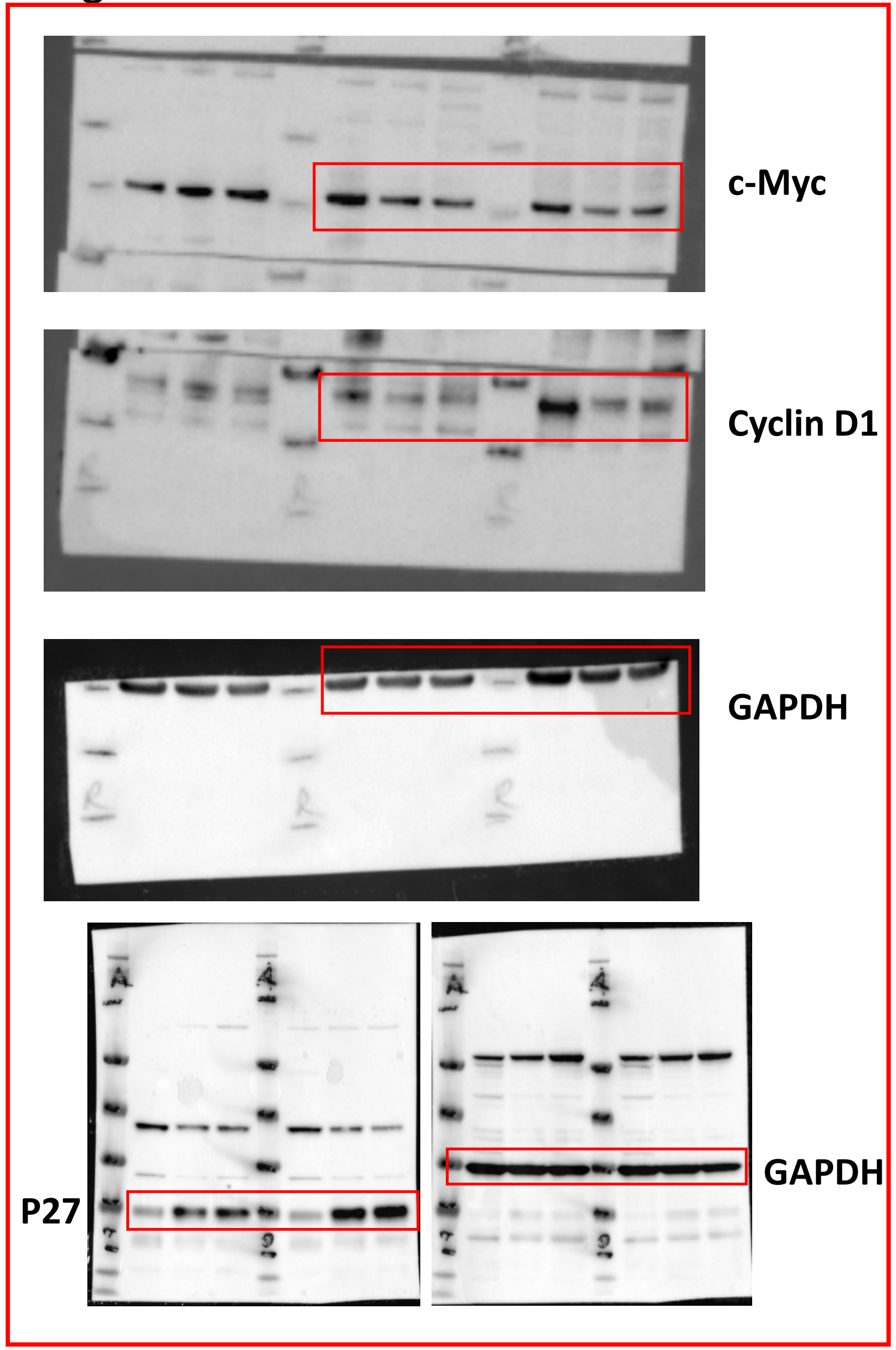

Figure 4E

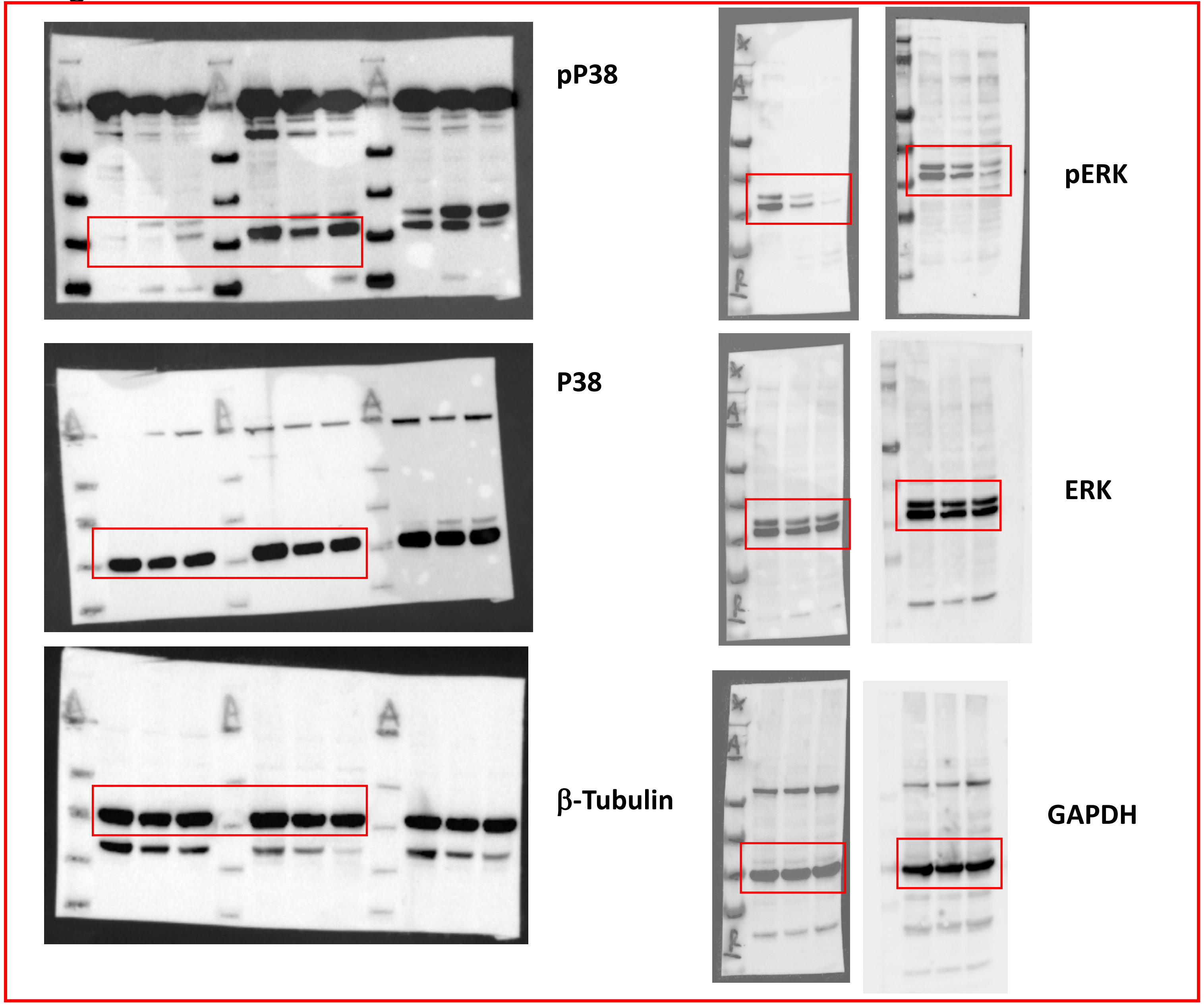

Figure 5D

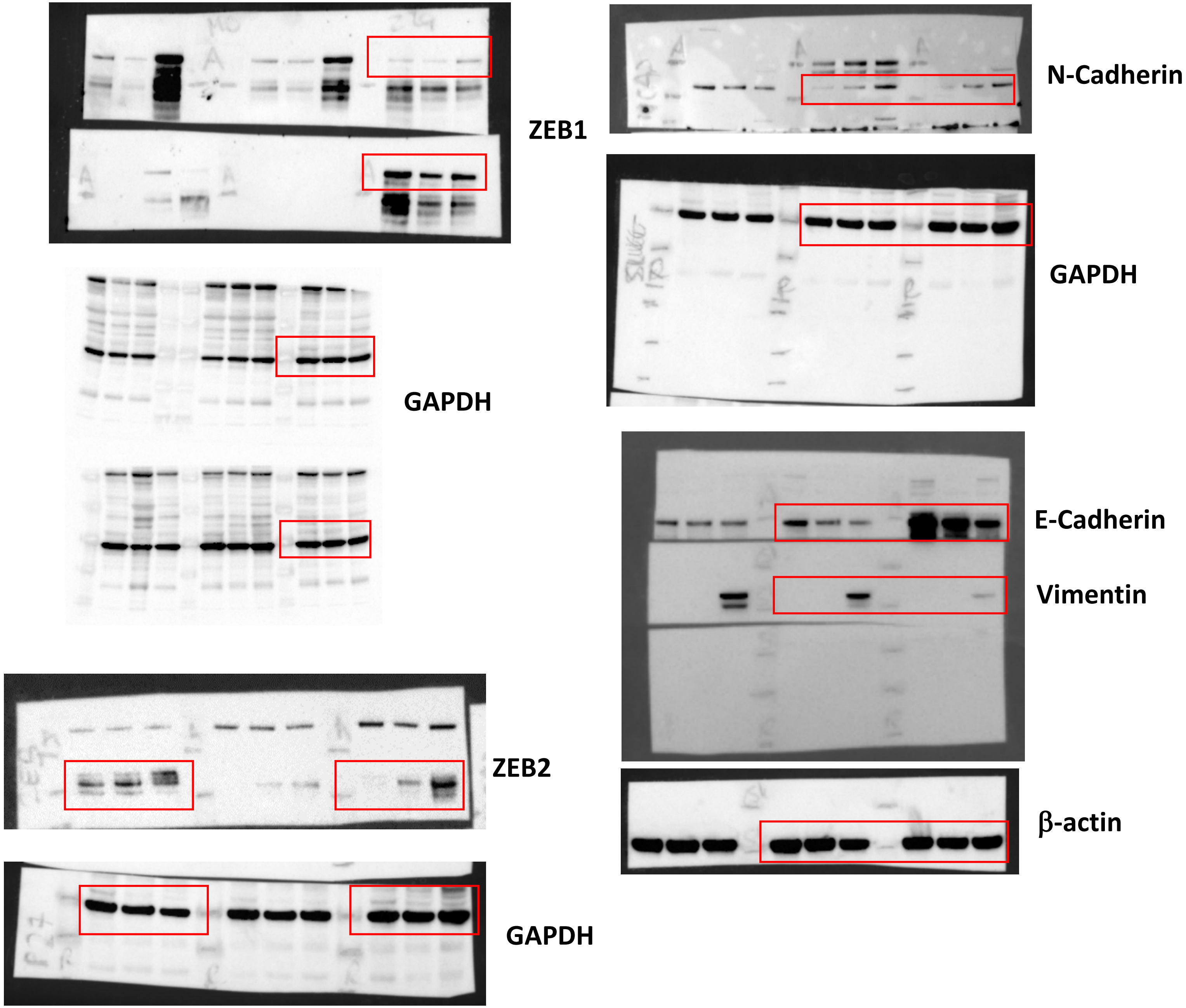
ZEB1
GAPDH
ZEB2
GAPDH
N-Cadherin
GAPDH
E-Cadherin
Vimentin
β-actin

Figure 6D

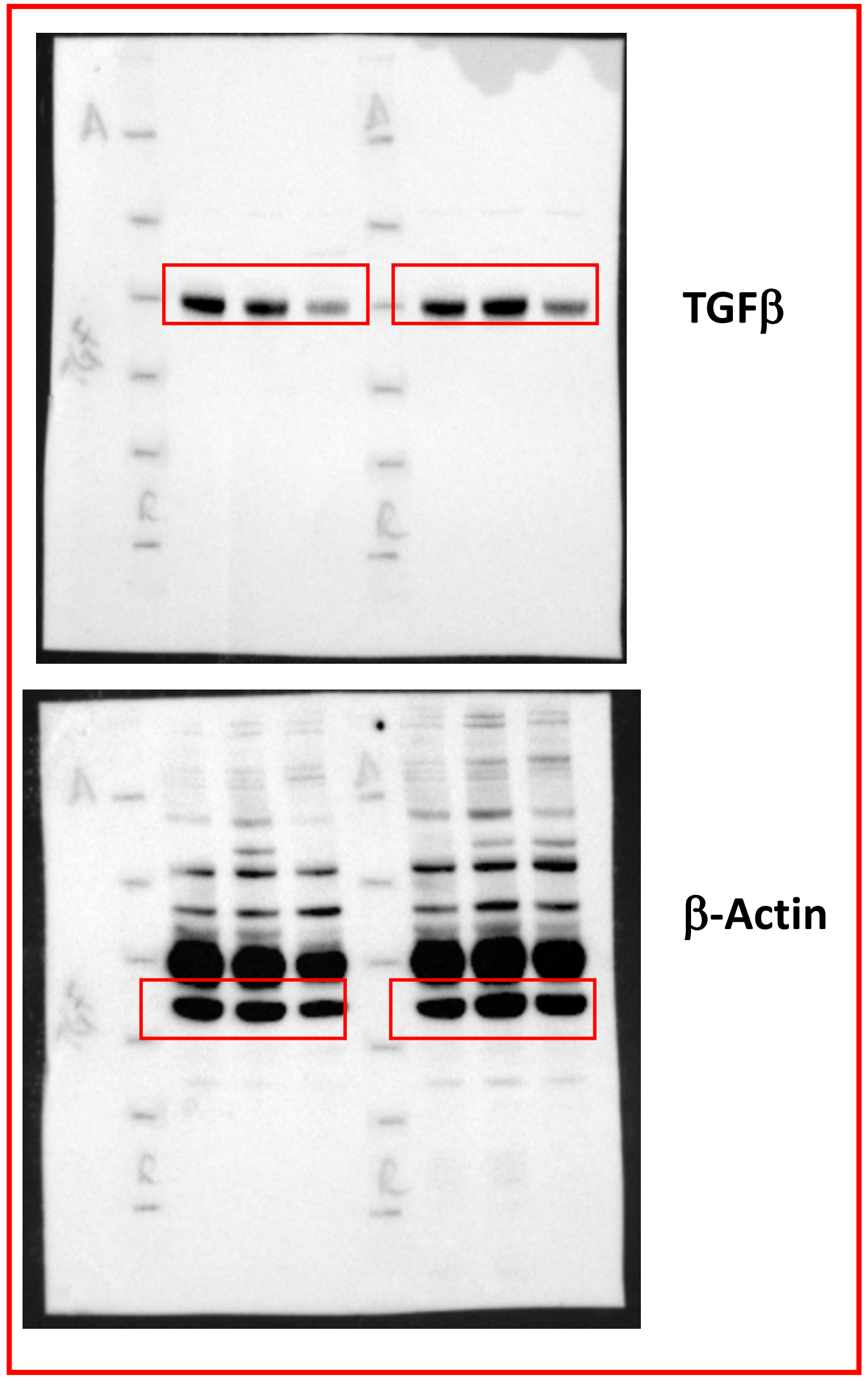

Figure 6E

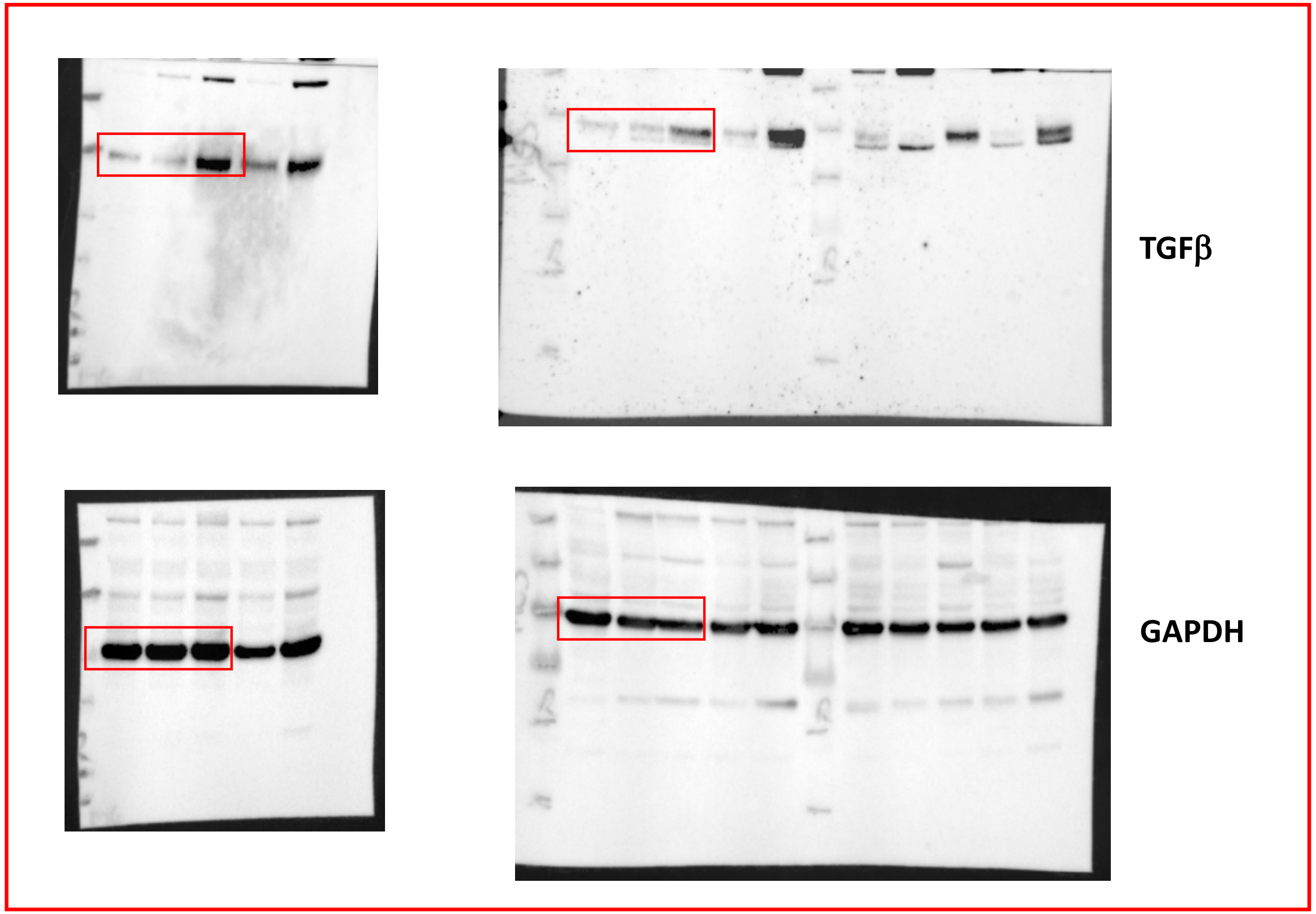

Figure 7B

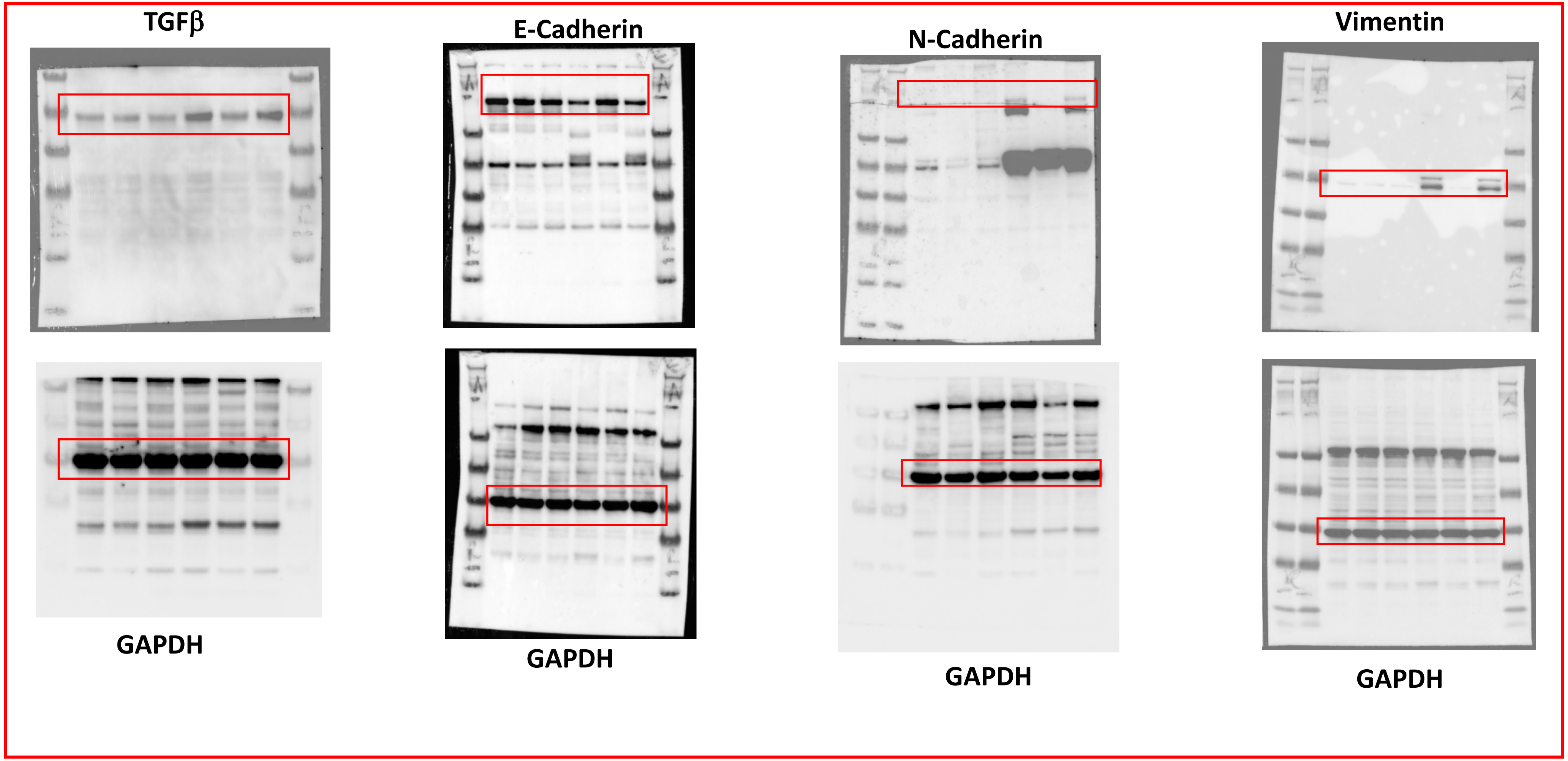

Figure 7C

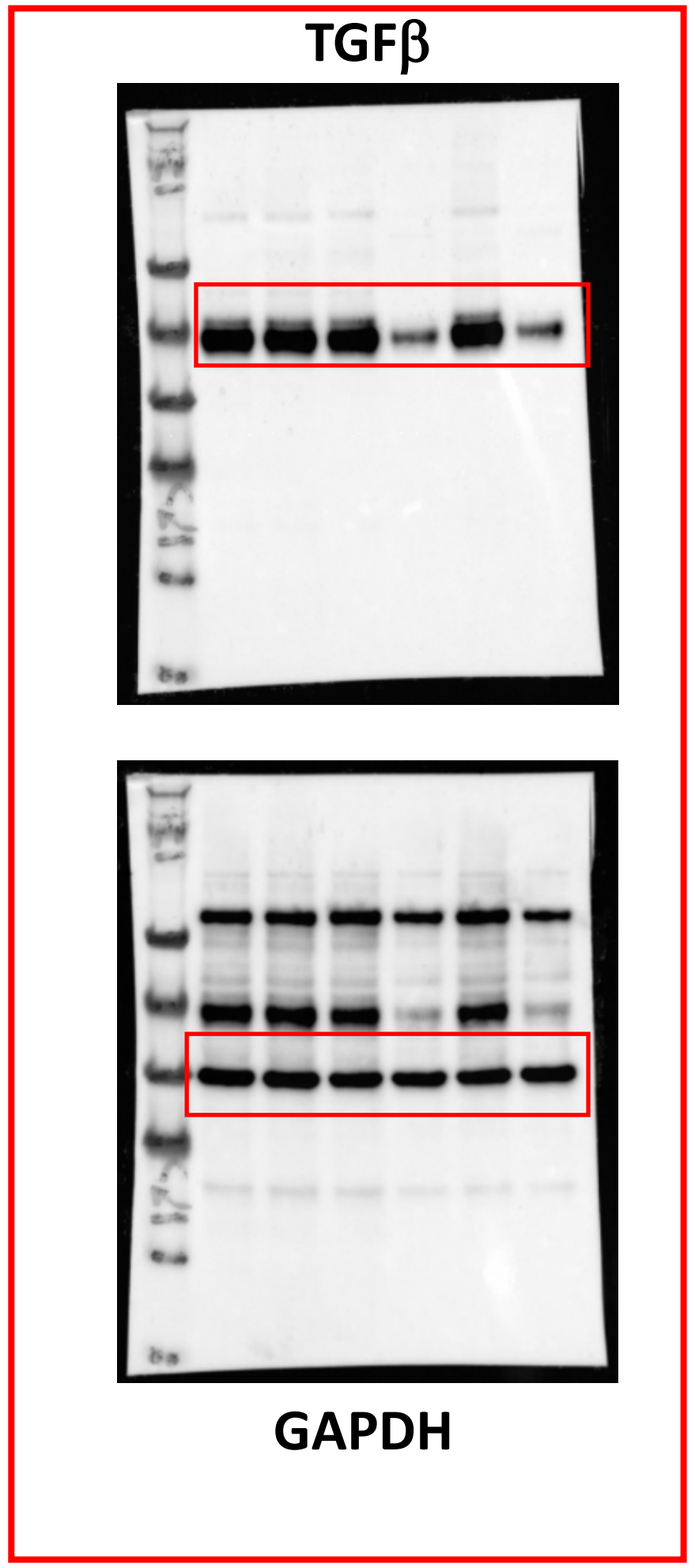

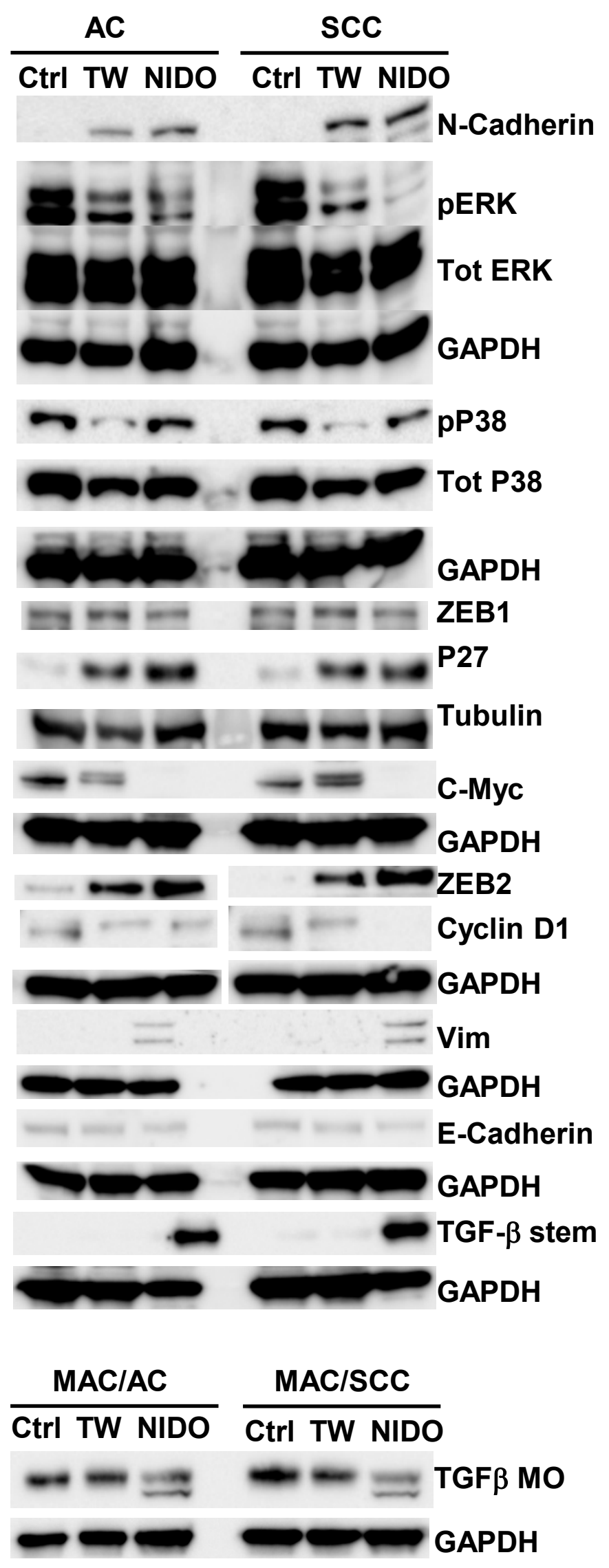
AC
SCC
Ctrl TW NIDO
Ctrl TW NIDO
N-Cadherin
pERK
Tot ERK
GAPDH
pP38
Tot P38
GAPDH
ZEB1
P27
Tubulin
C-Myc
GAPDH
ZEB2
Cyclin D1
GAPDH
Vim
GAPDH
E-Cadherin
GAPDH
TGF-β stem
GAPDH
MAC/AC
MAC/SCC
Ctrl TW NIDO
Ctrl TW NIDO
TGFβ MO
GAPDH

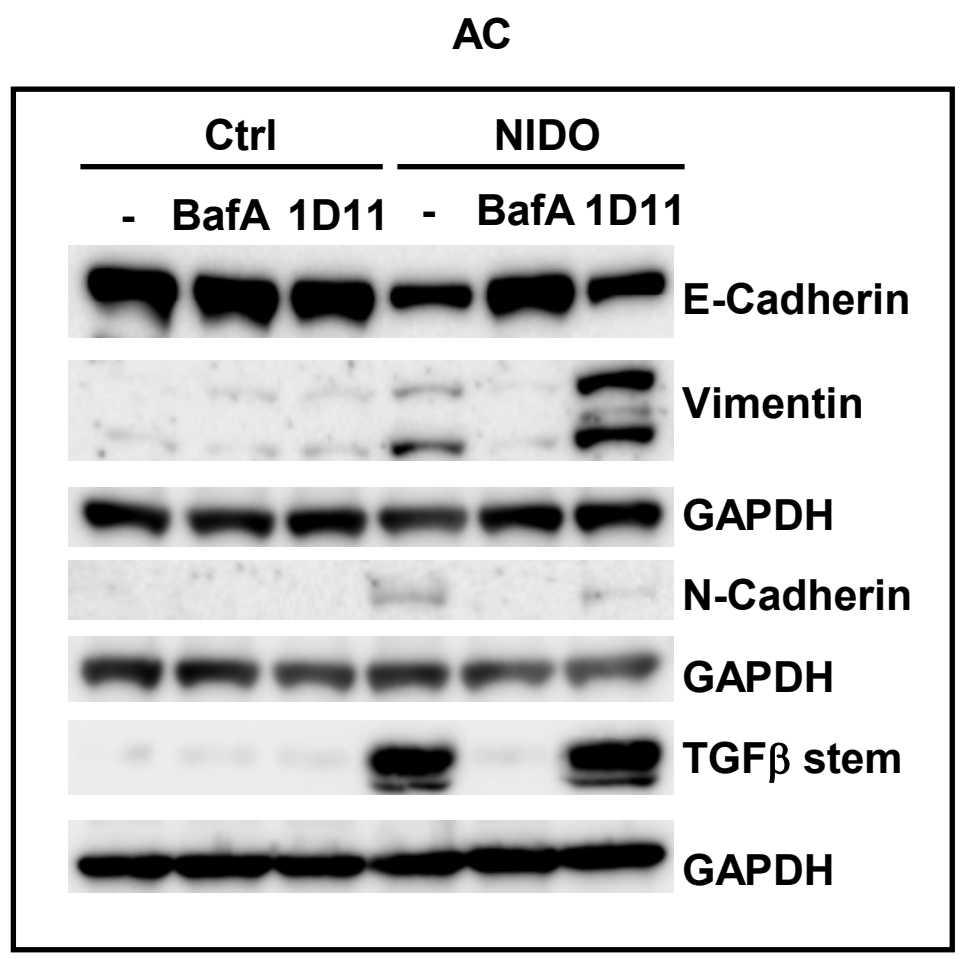
AC
Ctrl
NIDO
- BafA 1D11
- BafA 1D11
E-Cadherin
Vimentin
GAPDH
N-Cadherin
GAPDH
TGFβ stem
GAPDH

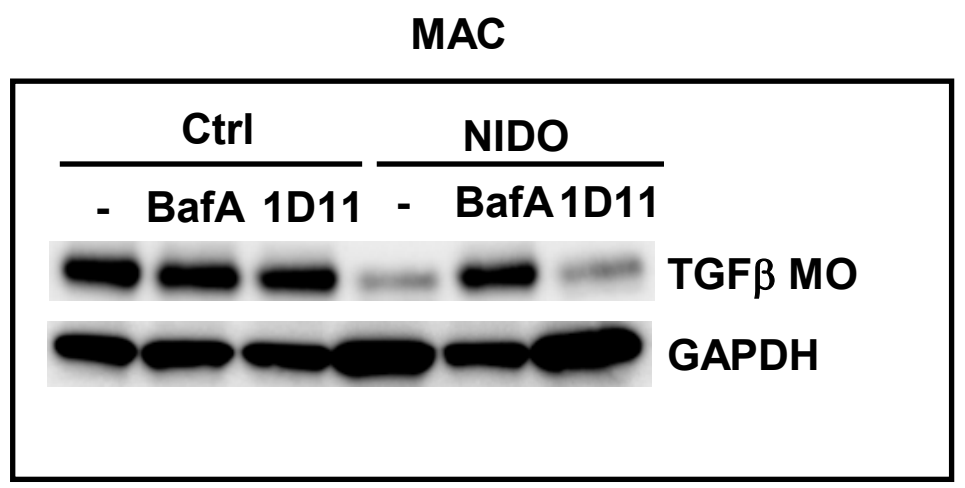
MAC
Ctrl
NIDO
- BafA 1D11
- BafA 1D11
TGFβ MO
GAPDH

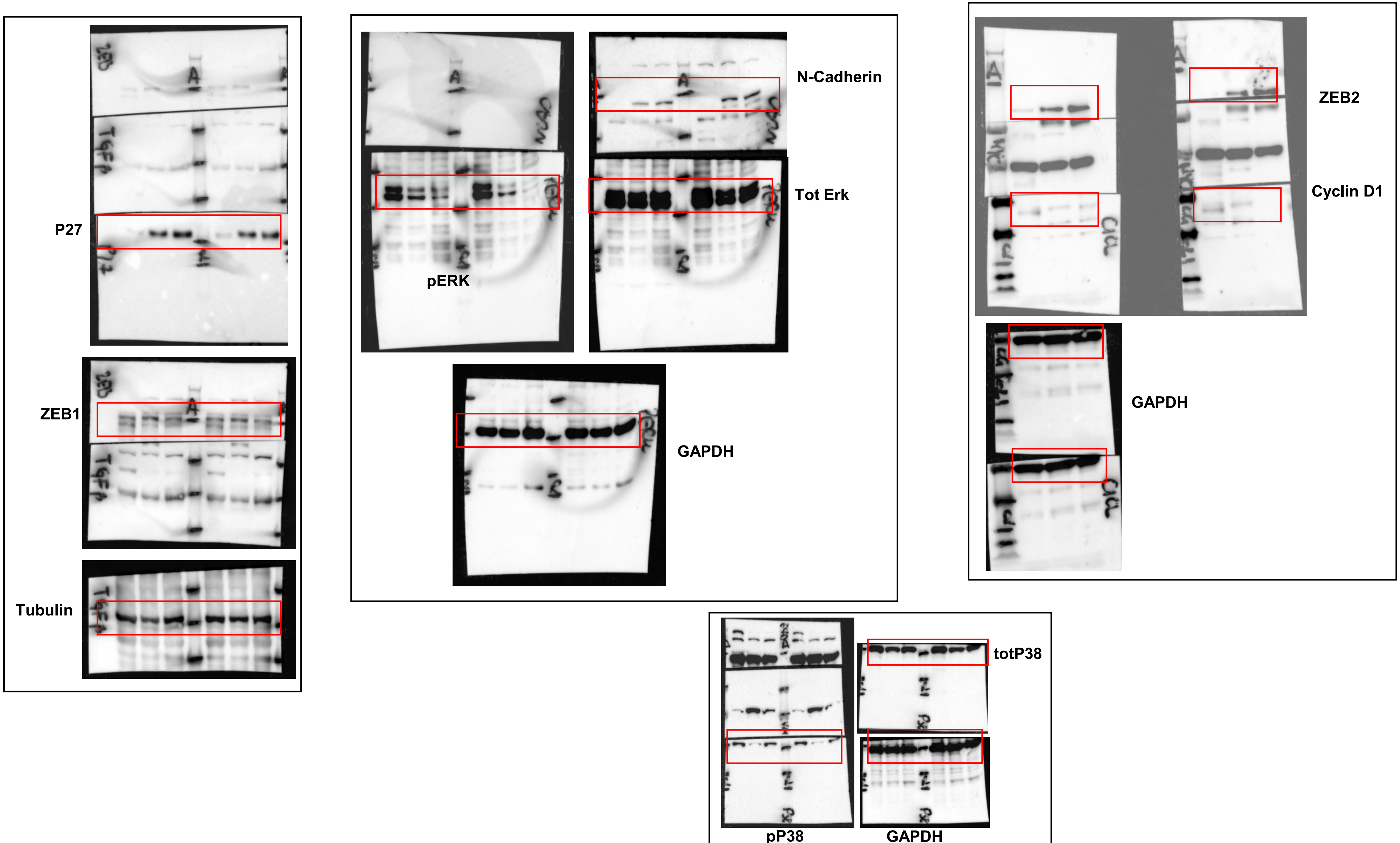
P27
ZEB1
Tubulin
N-Cadherin
Tot Erk
pERK
GAPDH
ZEB2
Cyclin D1
GAPDH
totP38
pP38
GAPDH

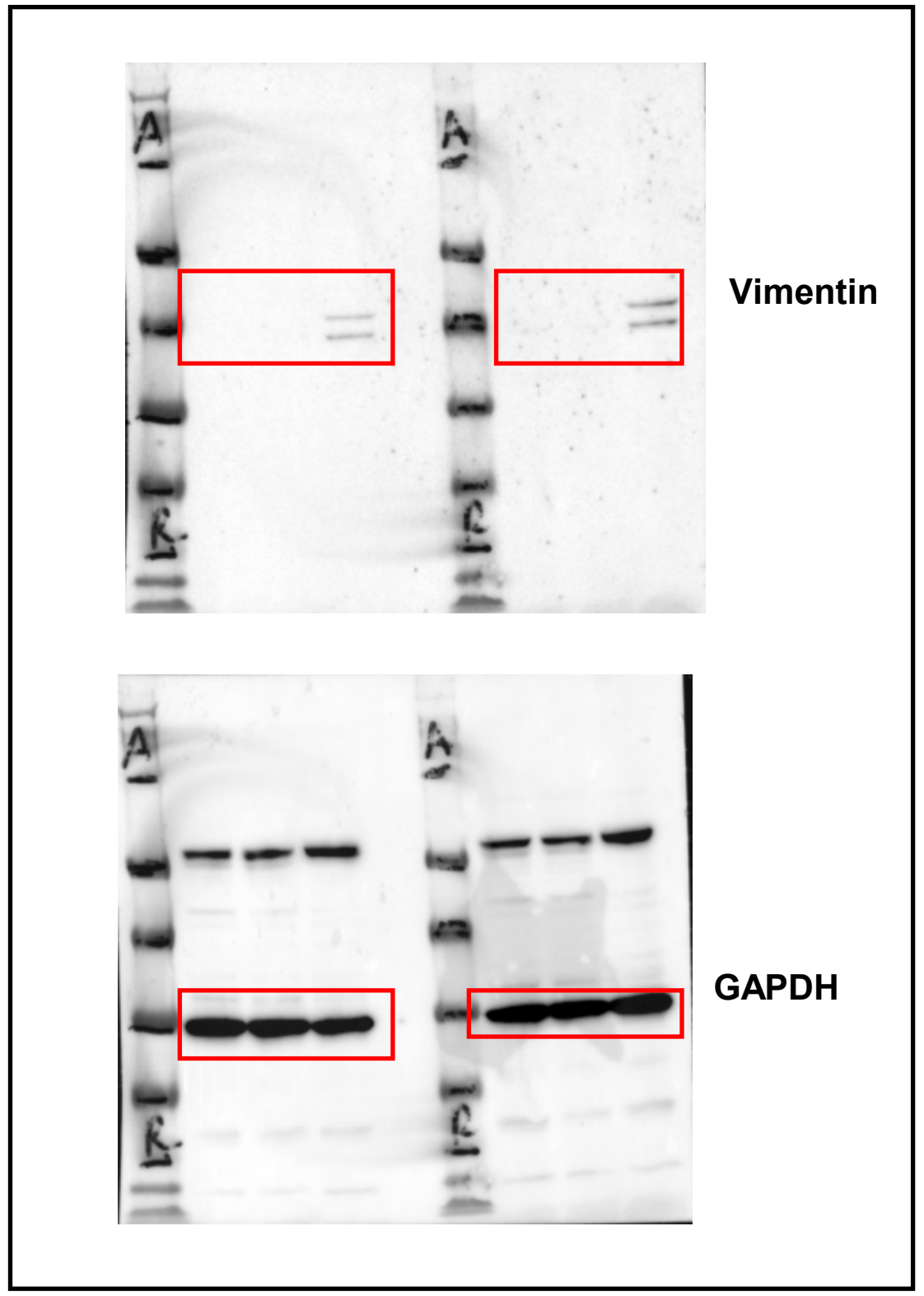
Vimentin
GAPDH

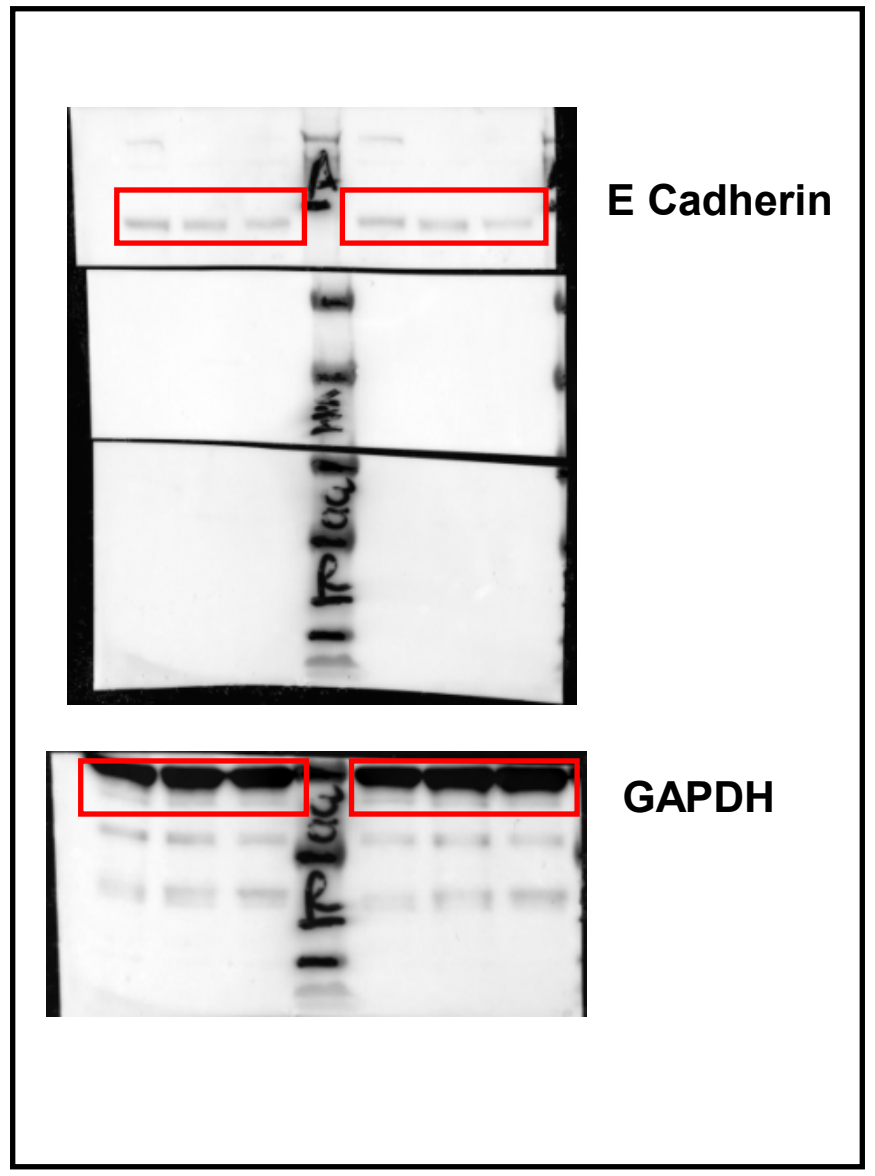
E Cadherin
GAPDH

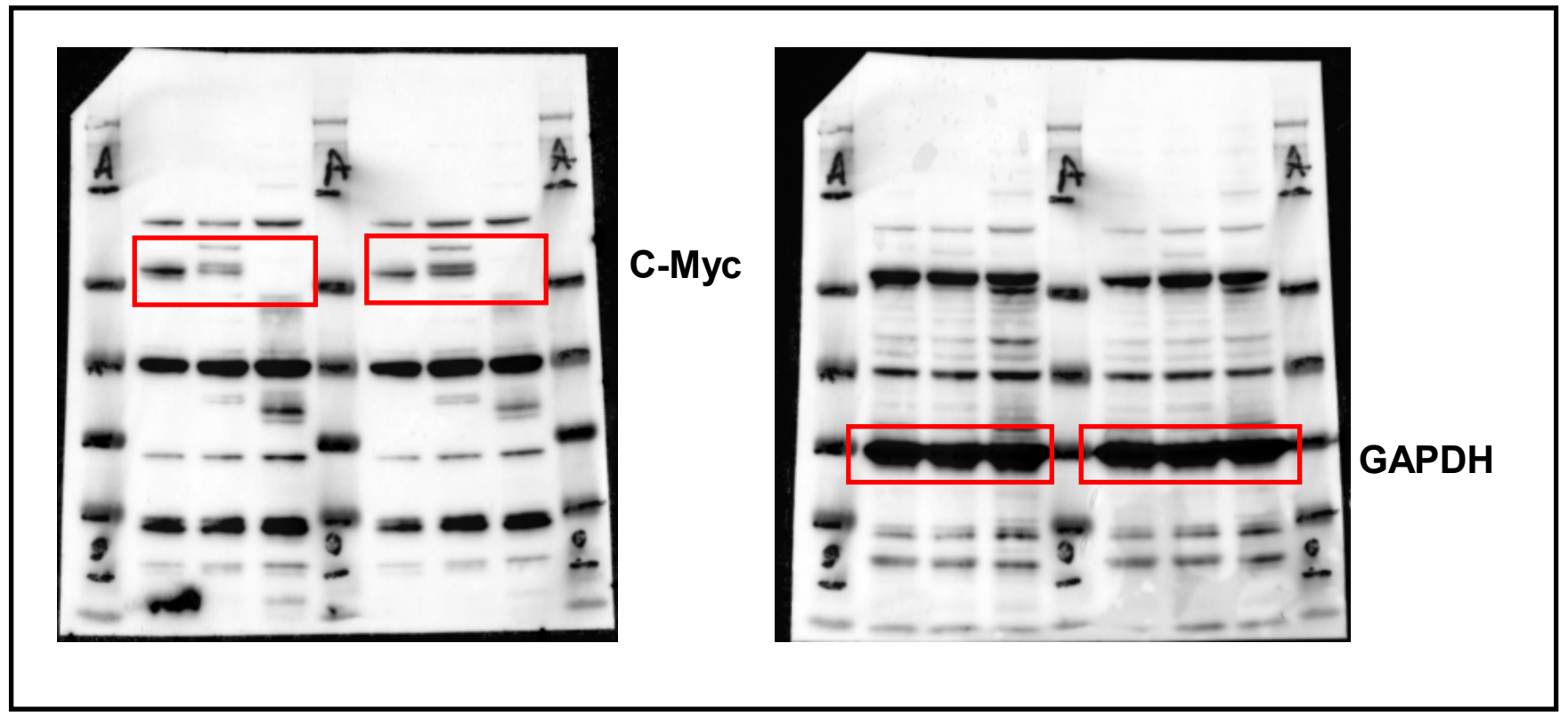
C-Myc
GAPDH

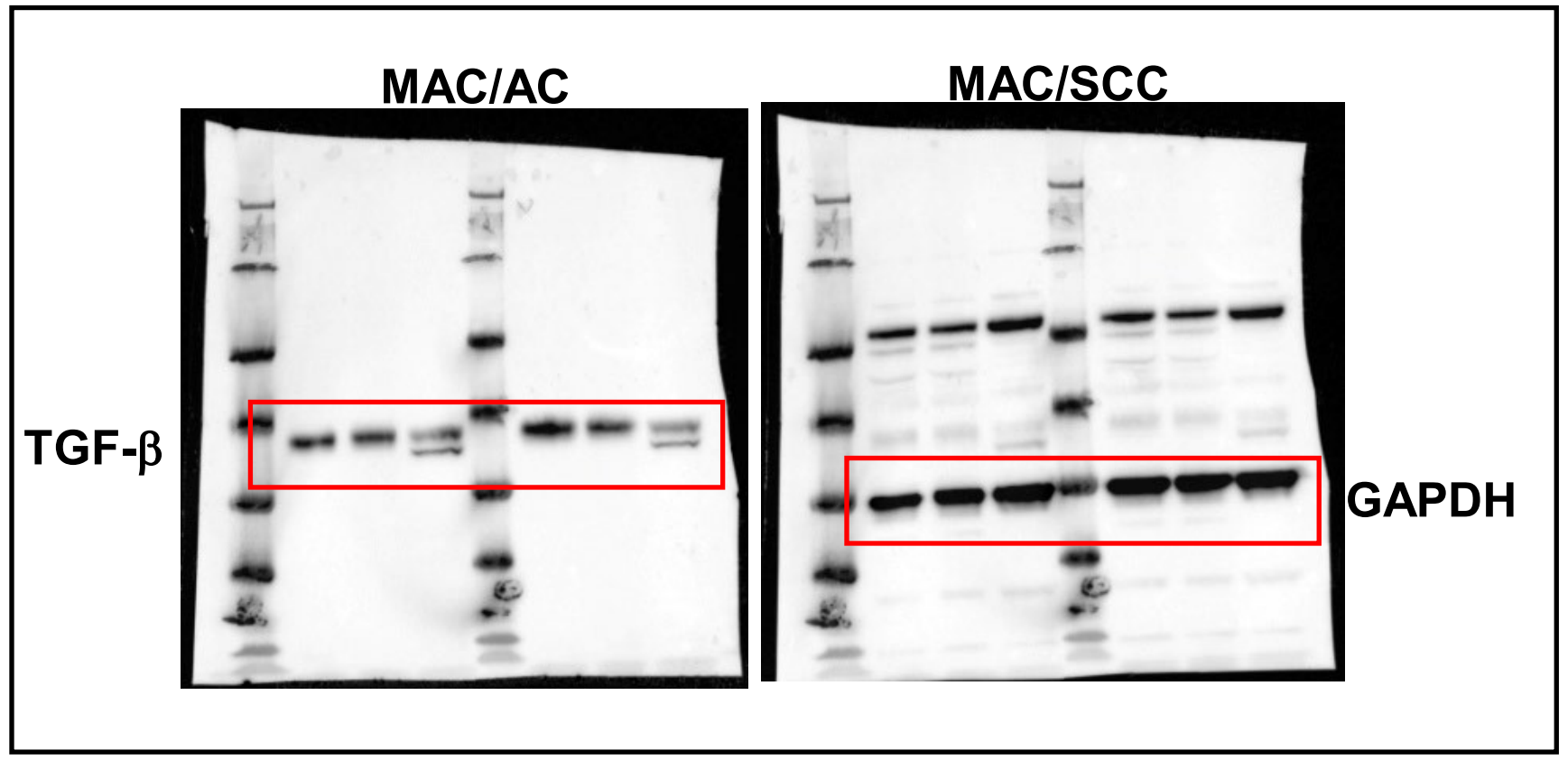
MAC/AC
MAC/SCC
TGF-β
GAPDH

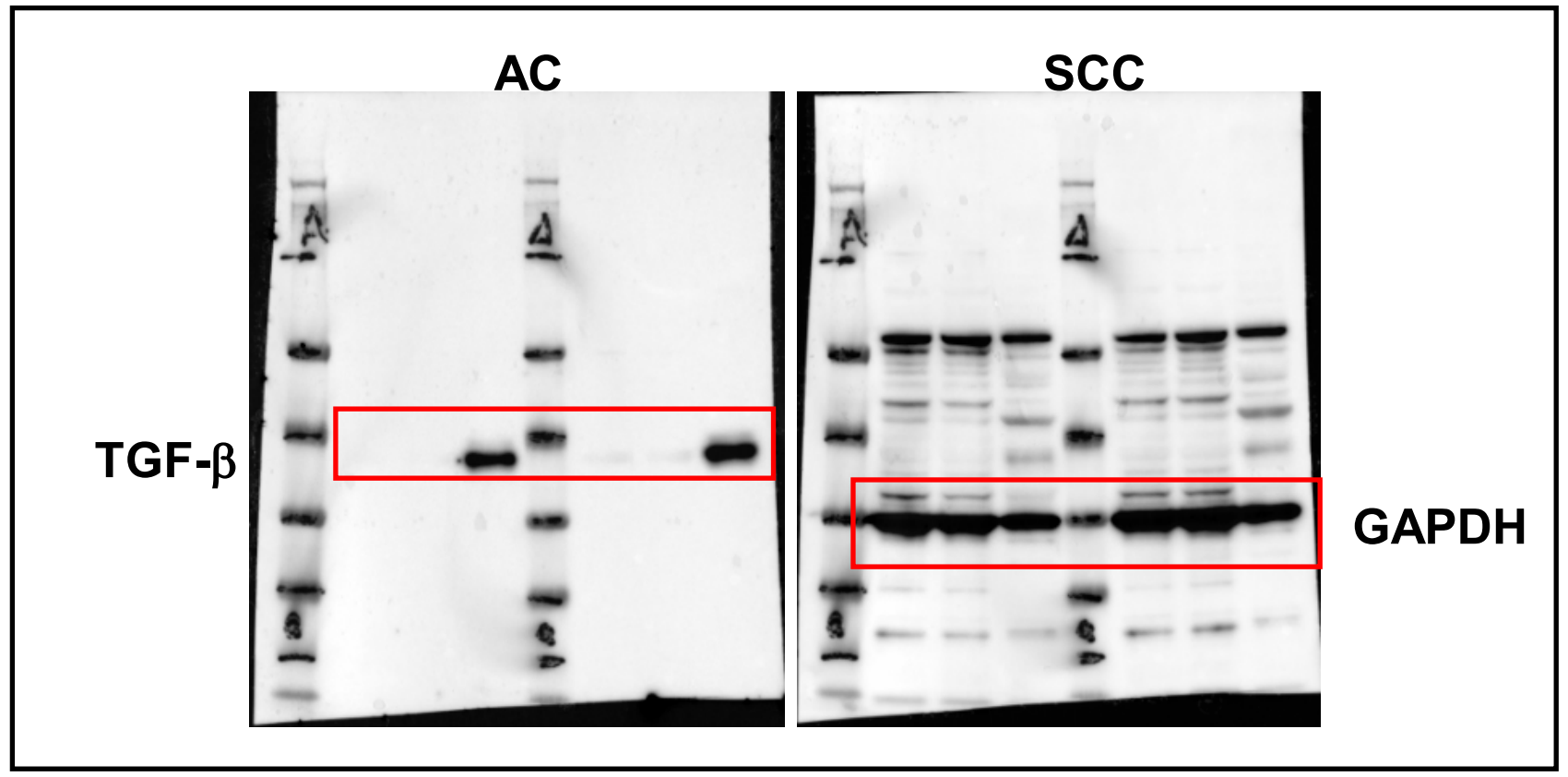
AC
SCC
TGF-β
GAPDH

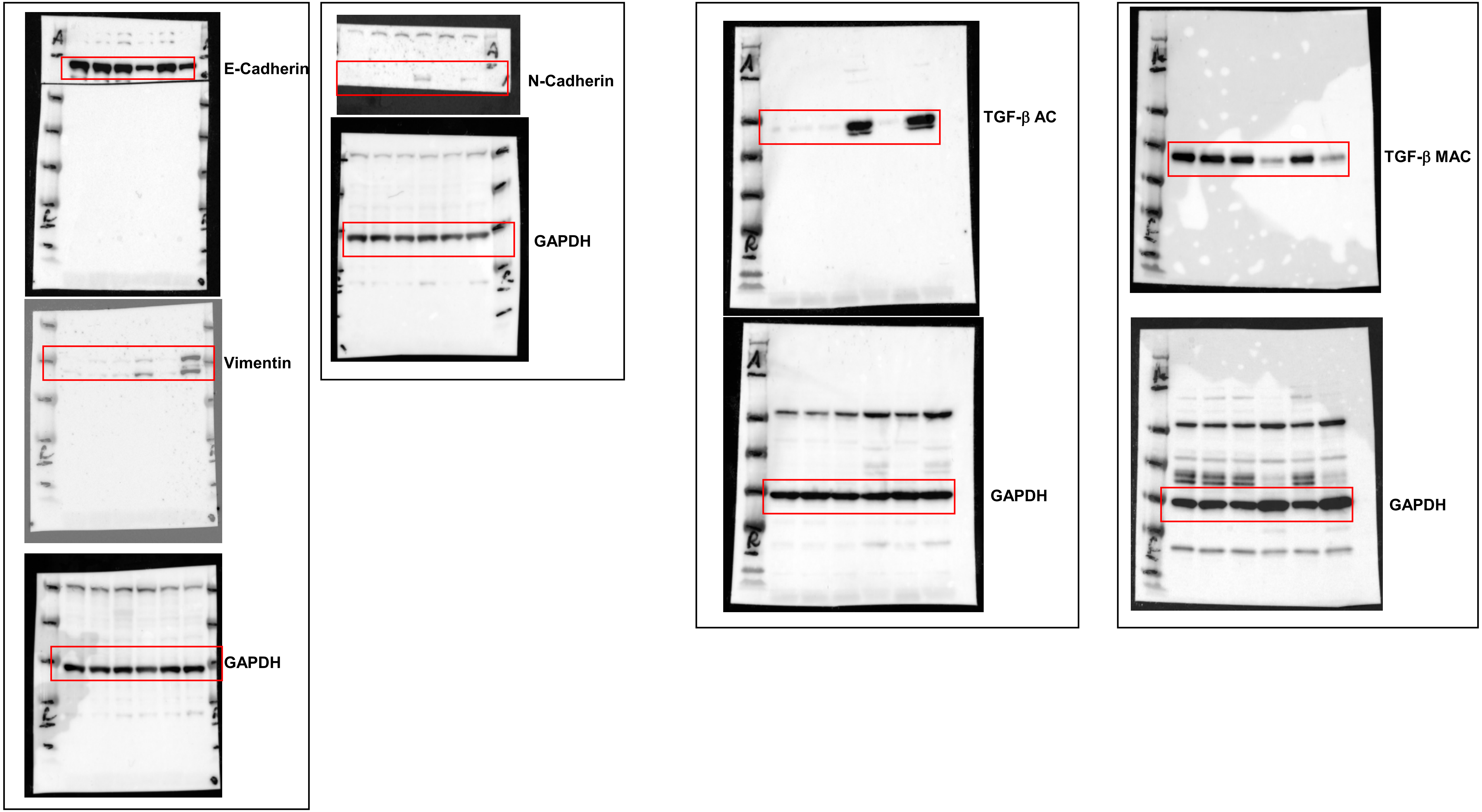
E-Cadherin
Vimentin
GAPDH
N-Cadherin
GAPDH
TGF-β AC
GAPDH
TGF-β MAC
GAPDH
